# Phytochemical Characterization and Integrated Biological Evaluation of *Baccharis dracunculifolia* DC Ethanol Extract: Antioxidant, Anti-Inflammatory and Toxicological Evidence

**DOI:** 10.3390/ph19101552

**Published:** 2026-09-30

**Authors:** Gustavo Ratti da Silva, Lucas Pires Guarnier, Arianne Jung Kluck, Mayara Alves Amorim, Arisa Namie Higashijima, Rafaela Caroline Santa Clara-Amorim, Julia Amanda Rodrigues Fracasso, Jaqueline de Bortoli Shirabayashi, Gabriel Augusto Rodrigues Beirão, Lucinéia dos Santos, Alan de Almeida Veiga, João Tadeu Ribeiro-Paes, Zilda Cristiani Gazim, Lauro Mera de Souza, Francislaine Aparecida dos Reis Lívero

**Affiliations:** 1 Laboratory of Preclinical Research of Natural Products, Post Graduate Program in Animal Science with Emphasis on Bioactive Products, Paranaense University, Umuarama 87502-210, Brazil; gustavoratti@gmail.com (G.R.d.S.); jaquebortoli@hotmail.com (J.d.B.S.); 2 Department of Genetic, Ribeirão Preto Medical School, University of São Paulo, Ribeirão Preto 14049-900, Brazil; lucasguarnier@usp.br; 3 Laboratory of Cardiometabolic Pharmacology, Federal University of Paraná (UFPR), Curitiba 81531-980, Brazil; ariannejung1@gmail.com (A.J.K.); arisahigashijima@ufpr.br (A.N.H.); rafaelacaroline@ufpr.br (R.C.S.C.-A.); 4 Department of Pharmacology, Federal University of Paraná (UFPR), Curitiba 81531-980, Brazil; mayara.amorim@ufpr.br; 5 School of Dentistry, São Paulo State University (UNESP), Araçatuba 16015-050, Brazil; j.fracasso@unesp.br (J.A.R.F.); lucineia.santos@unesp.br (L.d.S.); 6 Chemistry Laboratory of Natural Products, Graduate Program in Animal Science and Biotechnology Applied to Agriculture, Paranaense University, Umuarama 87502-210, Brazil; gabriel.beirao@edu.unipar.br (G.A.R.B.); cristianigazim@prof.unipar.br (Z.C.G.); 7 Instituto de Pesquisa Pelé Pequeno Príncipe, Faculdades Pequeno Príncipe, Curitiba 80250-060, Brazil; alanwessels@hotmail.com (A.d.A.V.); lauro.bioq@gmail.com (L.M.d.S.); 8 Department of Biotechnology, São Paulo State University, São Paulo 19806-900, Brazil; jtrpaes@yahoo.com.br

**Keywords:** acute oral toxicity, bioactive metabolites, clerodane diterpenes, phenolic acids, phenolic compounds, secondary metabolites

## Abstract

**Background/Objectives**: *Baccharis dracunculifolia* DC. is a medicinal plant and the primary botanical source of Brazilian green propolis. This study aimed to characterize the phytochemical profile of its ethanol extract and evaluate its antioxidant, anti-inflammatory, cytotoxic, and acute oral toxicity profiles. **Methods**: The extract was characterized by ultra-high-performance liquid chromatography coupled with quadrupole time-of-flight mass spectrometry (UHPLC-QTOF-MS). Antioxidant and anti-inflammatory activities were evaluated by complementary in vitro assays, cytotoxicity in NIH/3T3 fibroblasts, and acute oral toxicity according to OECD Guideline 423 in Wistar rats at 300 and 2000 mg/kg. **Results**: UHPLC-QTOF-MS analysis resulted in the tentative annotation of 54 metabolites, including phenolic acids, flavonoids, caffeoylquinic acid derivatives, diterpenes, glycolipids, and saponins. The extract showed antioxidant activity, with a DPPH IC_50_ of 0.77 ± 0.009 mg/mL and FRAP reducing capacity of 1.24 ± 0.22 µM ferrous sulfate/mg. Anti-inflammatory activity increased with concentration, reaching 58.60 ± 0.23%, 60.84 ± 0.40%, and 57.89 ± 2.96% inhibition of hemolysis, macrophage spreading, and phagocytosis, respectively, at 600 µg/mL (*p* < 0.05 vs. negative control). NIH/3T3 viability remained above 70% at all tested concentrations and exposure times. No mortality, treatment-related clinical signs, or significant changes in body weight, hematological and biochemical parameters, relative organ weights, or histopathological findings were observed at doses up to 2000 mg/kg. **Conclusions**: The *B. dracunculifolia* ethanol extract exhibited antioxidant and anti-inflammatory activities in vitro, without relevant cytotoxicity under the tested conditions, and showed no evidence of acute oral toxicity at doses up to 2000 mg/kg.

## 1. Introduction

The increasing interest in plant-derived natural products has intensified research into their chemical composition and biological activities, driven by the search for novel bioactive compounds with pharmaceutical and nutraceutical applications. Advances in phytochemical profiling have considerably expanded the characterization of specialized metabolites in medicinal plants, contributing to a better understanding of the relationship between chemical composition and biological effects. In this context, comprehensive phytochemical characterization has become an essential step in elucidating the biological potential of medicinal species and supporting their safe therapeutic and industrial applications [1].

The increasing use of plant-derived bioactive compounds in food systems has also raised important concerns regarding their safety for human consumption. Although natural products are often perceived as inherently safe, several studies have demonstrated that phytochemicals may exert toxic, genotoxic, or pro-oxidant effects depending on dose, exposure conditions, and chemical composition. In this context, the demand for safer and more sustainable alternatives has driven the search for bioactive ingredients derived from medicinal plants, particularly those intended for incorporation into functional foods and nutraceuticals. These products are frequently enriched with naturally occurring antioxidant and anti-inflammatory compounds, reflecting a broader shift toward preventive approaches to health and well-being [2,3]. Numerous studies have highlighted the potential of plant-derived compounds as key ingredients in the development of functional foods [4,5,6,7].

*Baccharis dracunculifolia* DC., a plant native to South America, has been extensively investigated for its antioxidant and anti-inflammatory properties, attracting interest in both traditional and scientific applications [8,9]. Commonly known as “vassourinha” or “alecrim-do-campo”, *B. dracunculifolia* belongs to the Asteraceae family and is the primary botanical source of resin used by *Apis mellifera* bees for the production of Brazilian green propolis [10,11,12]. Traditionally, *B. dracunculifolia* has been used in folk medicine in the form of aqueous preparations for the management of gastrointestinal disorders, including dyspeptic symptoms, as well as inflammatory conditions, fatigue, fever, and hypertension [13,14].

Phytochemical investigations of *B. dracunculifolia* have revealed a rich composition of flavonoids, terpenes, and phenolic acids, particularly caffeic and *p*-coumaric acids, which have been associated with its biological properties [14,15]. Recent advances in high-resolution mass spectrometry have further demonstrated the chemical diversity of this species, including glycosylated flavonoids, caffeoylquinic acid derivatives, diterpenes, and other specialized metabolites that may contribute to its pharmacological activities. Therefore, comprehensive phytochemical profiling is fundamental to better characterize the chemical composition of *B. dracunculifolia* and to support the investigation of its relationship with biological effects.

Several in vitro studies have demonstrated antioxidant [16], antibacterial [15], hepatoprotective [17], antifungal, antiparasitic [18], antiviral [19], immunomodulatory, and anti-inflammatory activities [20]. Experimental in vivo studies have also reported anti-inflammatory [21], antiulcerogenic [22], hepatoprotective [23], cardiometabolic [24], antigenotoxic, and antimutagenic activities [25]. These findings indicate a broad pharmacological potential of *B. dracunculifolia*, particularly in relation to inflammatory and gastrointestinal conditions, although the available evidence remains predominantly preclinical. Despite this pharmacological potential, evidence regarding its toxicological safety remains limited. Moreover, relatively few studies have integrated detailed phytochemical characterization with complementary biological and toxicological evaluations, limiting the understanding of how the chemical composition of the extract may contribute to its biological activities and safety profile.

Although the pharmacological potential of *B. dracunculifolia* has been extensively documented, its use as a source of bioactive compounds requires careful evaluation of both its phytochemical composition and toxicological profile. Considering that Brazilian green propolis, derived primarily from this species, is widely used in dietary supplements and functional products, investigating the chemical composition and safety of the plant extract itself is particularly relevant. The complex chemical composition of botanical extracts may influence both their biological effects and safety, highlighting the need for comprehensive phytochemical characterization combined with biological and toxicological assessment.

Therefore, the present study adopted an integrated approach combining untargeted ultra-high-performance liquid chromatography coupled with quadrupole time-of-flight mass spectrometry (UHPLC-QTOF-MS) phytochemical profiling with in vitro and in vivo assays to characterize the metabolite composition of an ethanol extract of *B. dracunculifolia* and evaluate its antioxidant, anti-inflammatory, cytotoxic, and acute oral toxicity profiles. Although previous studies have investigated the phytochemical composition and individual biological activities of *B. dracunculifolia*, comprehensive studies integrating chemical profiling with complementary biological and toxicological assessments remain limited. By combining these approaches, the present study provides a broader characterization of the biological activity and acute safety profile of the extract, contributing to the current understanding of the pharmacological potential of this species.

## 2. Results

### 2.1. UHPLC-QTOF-MS Profiling of Baccharis dracunculifolia DC

*B. dracunculifolia* contains a wide variety of natural compounds, including phenolic compounds, flavonoids, and terpenes [26]. These compounds are frequently associated with several pharmacological properties, making *B. dracunculifolia* an important source of phytochemicals. In our analyses, using liquid chromatography and mass spectrometry in positive- and negative-ion modes, with the best results from negative-ion detection, we identified several metabolites, confirming this plant as a rich source, especially of phenolic compounds. A total of 54 metabolites were tentatively annotated, confirming the chemical diversity of the extract, with a predominance of phenolic compounds.

**Figure 1:**
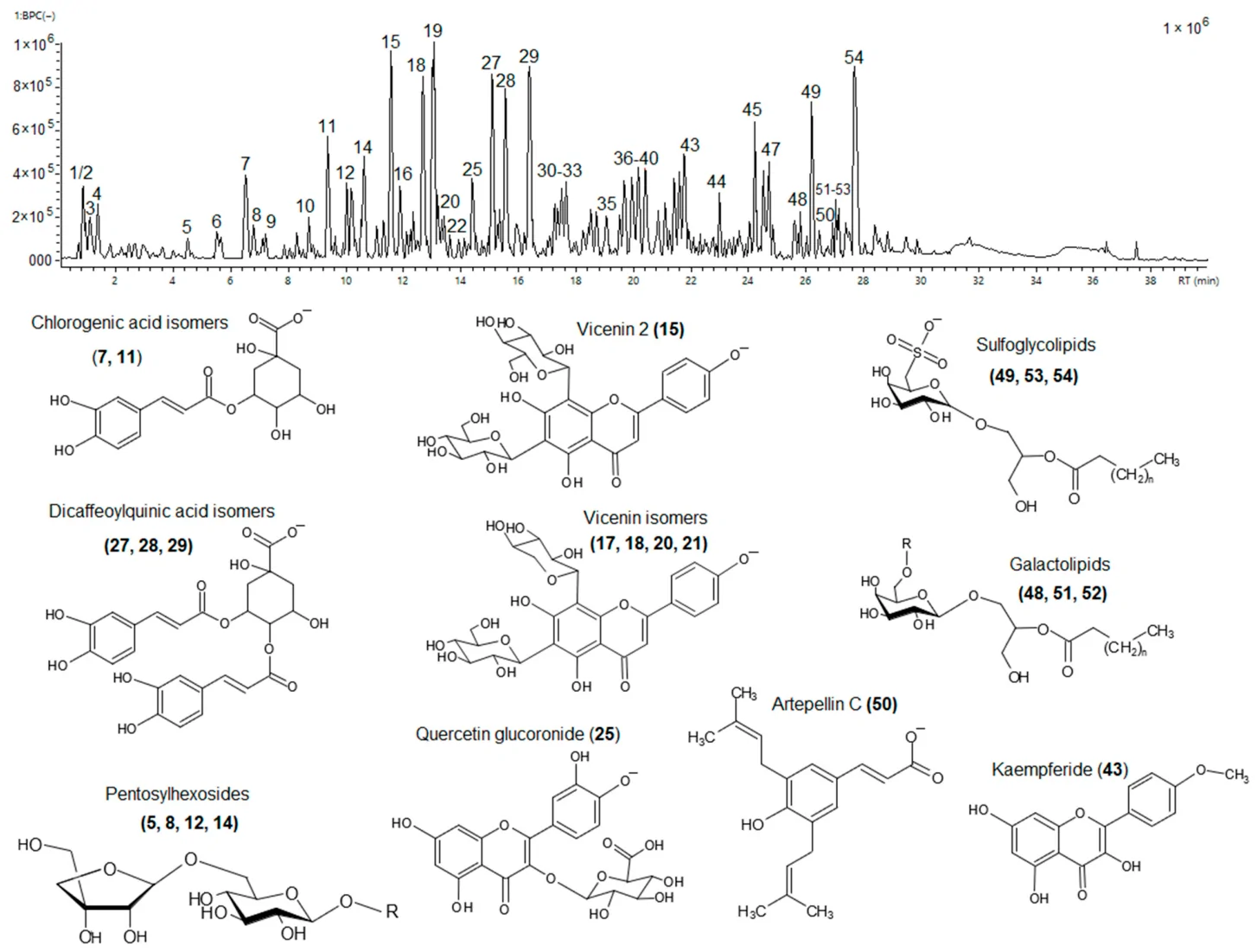
Phytochemical profile of the *Baccharis dracunculifolia* DC. extract obtained by UHPLC/QTOF-MS. Numbered peaks correspond to the tentatively identified metabolites, including pentosehexoses (5, 8, 12, 14), chlorogenic acid isomers (7, 11), vicenin-2 (15), vicenin isomers (17, 18, 20, 21), quercetin glucuronide (25), dicaffeoylquinic acid isomers (27–29), kaempferide (43), galactolipids (48, 51, 52), sulfoquinovosyl glycolipids (49, 53, 54), and artepillin C (50).

Polar compounds, such as gluconic acid (**1**), quinic acid (**2**), isocitric acid (**3**), and citric acid, were found (Figure 1) alongside a less common dimethyl citrate (**5**), appearing at *m*/*z* 219.0502 and fragments at *m*/*z* 111.008, 87.008, 67.018, consistent with the proposed structure as identified from other sources [27].

We also identified glycosides not previously reported in this plant. Compound **5**, at *m*/*z* 353.1448 [M-H]- and fragments at *m*/*z* 221.103 and 161.045. The neutral loss of 132.04 is consistent with the loss of a pentose residue, such as the most common xylose or arabinose, whereas *m*/*z* 161.045 is consistent with a hexose fragment, such as glucose. The fragment at *m*/*z* 221.103 was consistent with a hexose linked to a propyl radical; therefore, this compound is consistent with a propyl pentosylhexoside. Although we could not rule out other isomers, two low-abundance ions at *m*/*z* 113.023 and 101.024 indicated the loss of a water molecule and a formaldehyde molecule (Supplementary Figure S1A), respectively, consistent with loss of the ring-external carbon, which exists only in the furanosyl ring of a pentose.

The common plant glycosides composed of pentose and hexose include primeveroside and vicianoside [28]. In both cases, the pentoses are in pyranosyl ring configurations, meaning they lack ring-external carbons, thus formaldehyde loss is impaired. Instead, internal ring cleavages are common and lead to a typical loss of 60 and 90 Da. Therefore, the probable candidates were the isomers acuminoside (β-D-apiofuranosyl-(1→6)-β-D-glucopyranose), or apiinibioside (β-D-apiofuranosyl-(1→2)-β-D-glucopyranose). To confirm this hypothesis, we hydrolyzed and derivatized the extract for monosaccharide analysis. Gas chromatography–mass spectrometry (GC-MS) analysis of the alditol acetate derivatives confirmed the presence of apiose in this extract, consistent with the previous report, as apiitol (i.e., resulting from monosaccharide reduction) undergoes incomplete acetylation at the tertiary alcohol group, yielding apiitol pentaacetate and apiitol tetraacetate [29]. The presence of an uncommon tertiary hydroxyl also yields a characteristic EI-MS spectrum, easily distinguishable from other pentoses (Supplementary Figure S2). Therefore, compound **5** was consistent with a propyl-acuminoside or the isomer propyl apiinibioside.

Other identified compounds gave ions consistent with a similar disaccharide linked to other aglycones, such as compound **8**, at *m*/*z* 447.1136, consistent with a dihydroxybenzoic acid acuminoside (or apiinibioside), with the fragment at *m*/*z* 315.073 resulting from the loss of a pentose residue and the fragment at *m*/*z* 153.018 and 152.011 (radical), arising from the dihydroxybenzoic acid moiety (Supplementary Figure S1B). The net charge on the acidic aglycone prevented ion formation from monosaccharide units. The compound **12**, at *m*/*z* 401.1444 [M-H]- was identified as benzyl acuminoside (or apiinibioside). Supporting fragments were observed at *m*/*z* 269.102 (loss of the pentose residue), *m*/*z* 293.088 (disaccharide residue), and *m*/*z* 161.045 (hexose residue ion). As in 5, the ions from pentofuranosyl were observed at *m*/*z* 113.023 and 101.023, indicating the presence of apiose (Supplementary Figure S1C). Compound **14**, *m*/*z* 381.1757 [M-H]-, was consistent with isopentyl acuminoside (or apiinibioside). Observed fragments included *m*/*z* 293.089 (disaccharide residue), *m*/*z* 249.134 (hexose linked to pentyl radical), *m*/*z* 161.045 (hexose residue ion), and *m*/*z* 113.023/101.023 from apiose (Supplementary Figure S1E), supporting the tentative identification.

Other O-glycosides with different glycan moieties were also encountered. Compound **9**, at *m*/*z* 315.1077, and fragments at 195.065, 151.075, 125.023, 83.013, was consistent with hydroxytyrosol-hexoside. Compound **10** was observed at *m*/*z* 417.1031, exhibiting a mass 30.01 Da lower than compound **8**. This mass difference corresponds to CH2O, which is consistent with the structural variation between hexose and pentose units. Furthermore, MS/MS fragmentation supported this structural relationship, as the fragment at *m*/*z* 285.062 also showed a 30.01 Da decrease relative to the *m*/*z* 315.073 fragment of compound **8**. Additionally, the diagnostic fragments at *m*/*z* 241.071 and 153.019/152.011 (radical) confirmed the presence of the dihydroxybenzoic acid aglycone moiety (Supplementary Figure S1D). Therefore, the mass differences and the fragmentation pathways strongly suggest that compound **10** is a dihydroxybenzoic acid dipentoside.

Flavonoid C-glycosides formed by apigenin were identified. The compound **15**, at *m*/*z* 593.1514, gave the typical fragments from internal monosaccharide ring cleavage, yielding losses of consecutive neutral losses (NL) of 90 and 120 Da. This compound was identified as vicenin 2 (Supplementary Figure S1G). Compound **24** at *m*/*z* 755.1820 gave fragments at *m*/*z* 593.150 (loss of 162.03) alongside *m*/*z* 179.034, consistent with caffeic acid. Other fragments were characteristic of vicenin 2; therefore, this compound was assigned as the caffeoyl ester of vicenin 2. Two vicenin isomers are well recognized; however, in our analyses, 4 compounds (**17**, **18**, **20**, and **21**) were found in this extract with an MS profile characteristic of these compounds, appearing at *m*/*z* 563.139 and exhibiting characteristic fragments, including neutral losses of 60, 90, and 120 Da (Supplementary Figure S1H) [30].

Although less abundant, flavonoids *O*-glycosides were identified. They appeared to be forming the main fragments at *m*/*z* 300.028/301.035 characteristic of quercetin. Compound **23**, at *m*/*z* 741.1871, gave the main fragment of quercetin, but other negative fragments were poorly informative. In positive ion mode, this compound gave an ion at *m*/*z* 743.2003 [M + H]^+^ and fragment at *m*/*z* 465.101, characteristic of quercetin hexoside [31]. This structure is consistent with a quercetin triglycoside, formed by a rhamnose, a pentose, and a hexose linked to aglycone. Compound **25**, at *m*/*z* 477.0667 and a fragment at *m*/*z* 301.035, whose neutral loss (176.032 Da) is consistent with uronic acid residue, was identified as quercetin glucuronide (Supplementary Figure S1F). Compound **26**, at *m*/*z* 463.0874, was consistent with quercetin hexoside.

Caffeoyl quinic acids were also detected. They appeared as mono-, di-, and tricaffeoyl derivatives. The compounds **7** and **11** appeared at *m*/*z* 353.087, with main fragments at *m*/*z* 191.055 (quinic acid) and *m*/*z* 179.034 (caffeic acid), confirming them as chlorogenic acid isomers (Supplementary Figure S1I). Compounds **27**, **28**, and **29**, at *m*/*z* 515.119, gave the main ions at *m*/*z* 353.087, 191.055, and 179.034, characteristic of positional isomers of dicaffeoylquinic acids (Supplementary Figure S1J). The typical distribution of these compounds, as previously reported, is 3,4-dicaffeoylquinic acid, 3,5-dicaffeoylquinic acid, and 4,5-dicaffeoylquinic acid, according to chromatographic behavior [32]. The tricaffeoylquinic acid (compound **38**) was found at *m*/*z* 677.1501, with characteristic fragments at *m*/*z* 515.119, 353.087, 335.076, 191.055, 179.034, and 173.045, consistent with this structure.

Various compounds have appeared in this analysis, including characteristic ions of acid terpenes. Compounds **30** and **34** at *m*/*z* 377.159, compounds **31–33** at *m*/*z* 379.175, compounds **36** and **37** at *m*/*z* 361.165, compounds **39** and **40** at *m*/*z* 363.181, and compounds **41** and **42** at *m*/*z* 345.133 shared similar fragmentation behaviors, with the first cleavages leading to the loss of water (NL 18.010) and CO_2_ (NL 43.989 Da). Clerodane diterpenoids based on the structure of hawtriwaic acid (C_20_H_28_O_4_) have been found in *B. dracunculifolia* [26], and the genus *Baccharis* shows a great variety of these compounds [33]. Therefore, this natural products class is the most probable identification for these compounds, as the mass differences can be easily explained by incorporating hydroxyl groups and unsaturation into a core structure based on hawtriwaic acid (Figure 1 and Supplementary Table S1).

Other typical compounds from *B. dracunculifolia* were also found in this extract, as compound **43**, at *m*/*z* 299.0559, and fragments at *m*/*z* 284.032, 255.029, 227.034, 212.047, 183.044, 136.987, typical from a methyl kaempferol (Supplementary Figure S1K), as in kaempferide, previously found in this plant, and the compound **50** at *m*/*z* 299.1658, and fragments at *m*/*z* 255.175, 244.109, 200.120, 185.096, 145.065, characteristic of Artepillin C (Supplementary Figure S1L), also identified in this plant species [34]. A non-reported saponin was found at *m*/*z* 925.4797 (compound **44**). The fragments at *m*/*z* 775.428 and 763.425 are from losses of a pentose and a hexose residue, respectively, indicating a branched pattern of these interglycosidic linkages to uronic acid. The fragment at *m*/*z* 613.374 was from the dehydrated residue of uronic acid linked to the triterpenoid, typical of this kind of saponin [35] and the fragment at *m*/*z* 455.351 is a characteristic ion from ursolic/oleanolic acids; therefore, compound **44** is consistent with a ursolic/oleanolic acid attached by a trisaccharide.

Glycolipids were also found in this extract. Compounds **48** and **52**, at *m*/*z* 721.3640 and 699.3799 [M+HCOO]-, gave typical fragments of neutral glycolipid, after loss of formiate adduct, at *m*/*z* 415.145 and 397.135 from a disaccharide linked to glycerol due to the loss of a fatty acid, *m*/*z* 179.055 and 161.046 from the hexoses. The compound **48** gave the ion at *m*/*z* 277.216, typical of linolenic acid (C_18:3_), whereas the compound **52** gave the ion at *m*/*z* 255.232, from palmitic acid (C_16:0_), thereby being identified as digalactosyl-monoacylglycerols, as they are typical of plants [36]. A monogalactosyl-monoacylglycerol (**51**) was also detected in this extract, appearing at *m*/*z* 559.311 [M+HCCO]-. The fragment at *m*/*z* 179.055 confirmed the presence of a hexose, and at *m*/*z* 277.215, the linolenic acid (C_18:3_) (Supplementary Figure S1M).

Alongside the neutral galactolipids, sufonolipids were also identified (compounds **49**, **53**, and **54**). All these lipids were identified as sulfoquinovosylmonoacylglycerol (SQMG), based on characteristic fragments of sulfoquinose at *m*/*z* 317.053, 299.043 (linked to glycerol), 225.007 from dehydrated sulfoquinovose, and 94.980 from methanolsulfonate [36]. The differences among these lipids are attributed to the fatty acids, identified from the fragmentation pattern as linolenic acid (*m*/*z* 277.216), linoleic acid (*m*/*z* 279.233), and palmitic acid (*m*/*z* 255.232) (Supplementary Figure S1N).

### 2.2. Total Phenolic and Flavonoid Contents in Baccharis dracunculifolia DC Extract

The total phenolic and flavonoid contents of the crude *B. dracunculifolia* extract were evaluated at different extract concentrations (0.25–1.00 mg/mL) (Table 1). The measured total phenolic content increased from 4.68 ± 0.67 µg gallic acid equivalents/mg of crude extract (GAE/mg CE) at 0.25 mg/mL to 72.56 ± 7.16 µg GAE/mg CE at 1.00 mg/mL, with significant differences among the concentrations. This increase reflects the response obtained with increasing extract concentration in the analytical assay and should not be interpreted as an enrichment of the extract in phenolic compounds. In contrast, the total flavonoid content showed a less pronounced variation among the tested concentrations, ranging from 472.00 ± 8.43 to 515.70 ± 20.30 µg quercetin equivalents/mg of crude extract (QE/mg CE). The highest value was observed at 0.25 mg/mL (515.70 ± 20.30 µg QE/mg CE), followed by 507.79 ± 4.08 µg QE/mg CE at 0.50 mg/mL, 472.00 ± 8.43 µg QE/mg CE at 0.75 mg/mL, and 495.90 ± 9.35 µg QE/mg CE at 1.00 mg/mL. According to Tukey’s test, the flavonoid content at 0.75 mg/mL was significantly lower than that at 0.25 and 0.50 mg/mL, whereas the value at 1.00 mg/mL did not differ significantly from the other concentrations.

### 2.3. Determination of Antioxidant Capacity of Baccharis dracunculifolia DC Extract

The in vitro antioxidant activity of the *B. dracunculifolia* extract is presented in Table 2. In the 2,2-diphenyl-1-picrylhydrazyl (DPPH) assay, the extract exhibited a half-maximal inhibitory concentration (IC_50_) of 0.771 ± 0.009 mg/mL, indicating moderate radical scavenging activity. For comparison, the positive control quercetin showed a much lower IC_50_ value of 0.010 ± 0.001 mg/mL, demonstrating stronger antioxidant activity. The ferric reducing antioxidant power (FRAP) assay revealed that the *B. dracunculifolia* extract had a reducing power of 1.244 ± 0.223 µM ferrous sulfate/mg, which was significantly lower than the positive control Trolox, which demonstrated a higher reducing capacity of 9.175 ± 0.001 µM ferrous sulfate/mg.

### 2.4. In Vitro Anti-Inflammatory Screening of Baccharis dracunculifolia DC

#### 2.4.1. Hemolysis Inhibition

**Figure 2:**
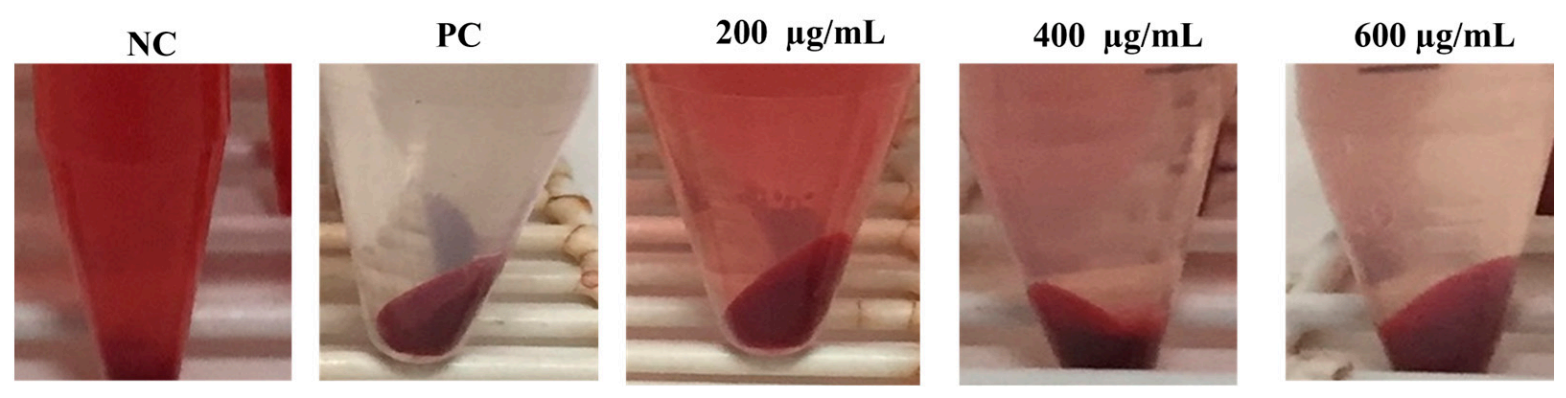
Representative images of the hemolysis inhibition assay. NC, negative control (0.9% physiological saline); PC, positive control (dexamethasone, 40 μg/mL); *B. dracunculifolia* extract, 200, 400, and 600 μg/mL.

Among the *B. dracunculifolia* concentrations evaluated, the highest anti-hemolytic response was observed at 600 µg/mL, with a percentage of hemolysis inhibition of 58.6 ± 0.23, indicating the most potent anti-hemolytic activity among the treatments tested. A concentration-dependent effect was evident, with increased anti-hemolytic activity corresponding to higher concentrations of the extract. Statistically significant differences were observed between all extract concentrations and the negative control (NC) (*p* < 0.05), further supporting the concentration-dependent nature of the extract’s efficacy in inhibiting hemolysis (Table 3). Representative images of the hemolysis inhibition assay are shown in Figure 2.

#### 2.4.2. Effect of *Baccharis dracunculifolia* Extract on Macrophage Spreading

**Figure 3:**
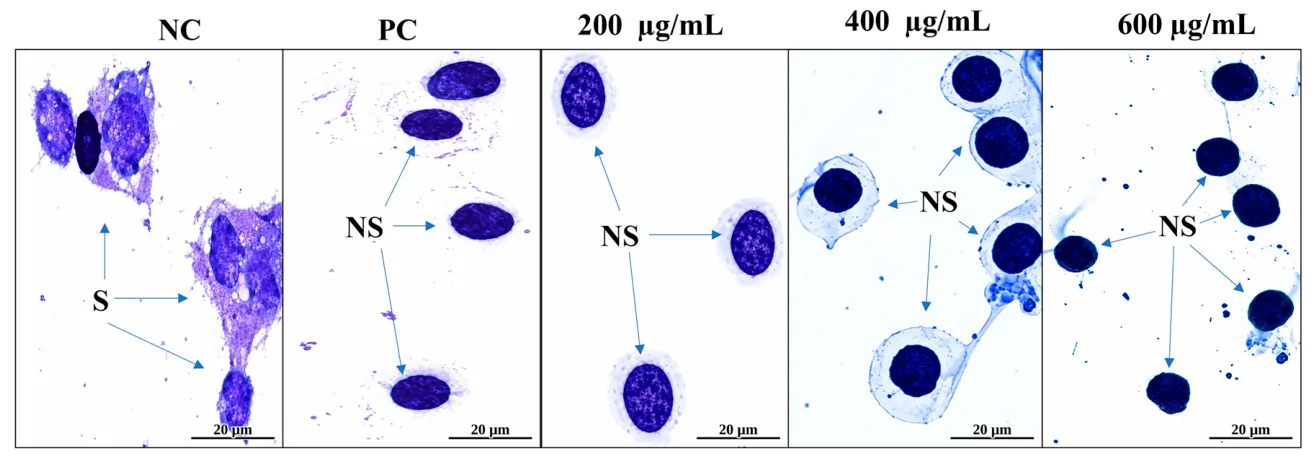
Representative photomicrographs of RAW 264.7 macrophages in the spreading inhibition assay. NC, negative control; PC, positive control; *B. dracunculifolia* extract (200, 400, and 600 μg/mL). S and arrows indicates spreading cells, whereas NS and arrows indicates non-spreading cells. Magnification: 400×. Scale Bar: 20 µm.

The *B. dracunculifolia* extract exhibited a concentration-dependent increase in the percentage of inhibition of spreading, with the highest inhibitory activity observed at 600 µg/mL (Table 4). Statistical analysis confirmed significant differences between all extract-treated groups and the NC (*p* < 0.05). Representative micrographs illustrating the morphological changes associated with macrophage spreading are presented in Figure 3.

#### 2.4.3. Inhibition of Phagocytosis

**Figure 4:**
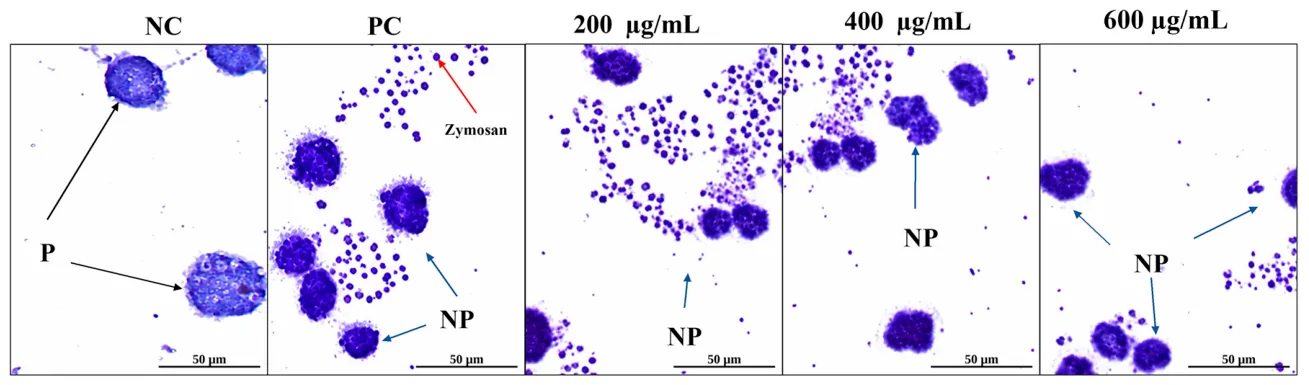
Representative photomicrographs of RAW 264.7 macrophages in the phagocytosis inhibition assay. NC, negative control; PC, positive control; 200, 400, and 600 μg/mL, cells treated with *B. dracunculifolia* extract at the indicated concentrations. Zymosan particles (insoluble cell-wall preparation from *Saccharomyces cerevisiae*) were used as the phagocytic target and are indicated by the red arrow. P and black arrows indicate phagocytic macrophages (cells with internalized zymosan particles); NP and blue arrows indicate non-phagocytic macrophages (cells without internalized particles). Original magnification: 400×. Scale bars: 50 μm.

Treatment with *B. dracunculifolia* extract led to a concentration-dependent increase in phagocytosis inhibition, with the highest inhibition observed at 600 µg/mL. Statistical analysis revealed significant differences between all extract concentrations and the NC (Table 5). Representative photomicrographs illustrating the effects of the extract on macrophage phagocytic activity are presented in Figure 4.

### 2.5. Safety Assessment of Baccharis dracunculifolia DC Extract

#### 2.5.1. Impact of *Baccharis dracunculifolia* on Fibroblast Cell Viability

Table 6 presents the results of the 3-(4,5-dimethylthiazol-2-yl)-2,5-diphenyltetrazolium bromide (MTT) cytotoxicity assay performed using NIH/3T3 mouse dermal fibroblasts to evaluate the effects of *B. dracunculifolia* extract on cell viability at different concentrations and exposure times. At concentrations of 100, 200, and 400 µg/mL, the extract showed minimal effects on cell viability, which remained close to 100% after 24 h, with increases of 4.98%, 2.19%, and 1.19%, respectively. At higher concentrations, cell viability remained above 70%, a commonly used threshold for cytotoxicity assessment. A concentration- and time-dependent reduction in cell viability was observed at 48 and 72 h, with progressively lower viability at higher extract concentrations and longer exposure times. Nevertheless, cell viability remained above 70% even after 72 h at the highest concentration tested. These findings indicate that the extract did not exhibit relevant cytotoxicity toward NIH/3T3 cells under the experimental conditions evaluated.

#### 2.5.2. Evaluation of Clinical Signs, Relative Organs Weight and Body Weight of Rats Treated with *Baccharis dracunculifolia* DC Extract

Animals treated orally with a single dose of *B. dracunculifolia* extract at 300 or 2000 mg/kg showed no treatment-related behavioral or physiological changes during the observation period, from 30 min to 14 days, compared with the basal group (Table 7). Body weight data are presented in Supplementary Table S2. Throughout the 14-day observation period, animals in all groups showed a progressive increase in body weight. The basal group exhibited a steady weight gain, reaching a final body weight of 236.30 ± 1.89 g on day 14. Similarly, both treatment groups showed progressive weight gain, with final body weights of 228.70 ± 3.77 g and 221.50 ± 4.26 g for the 300 and 2000 mg/kg groups, respectively. No significant differences in body weight gain were observed between the treated and basal groups, indicating that treatment with the extract did not adversely affect body weight during the observation period. Relative organ weights of the liver, spleen, heart, and kidneys were also evaluated. No statistically significant differences were observed between either treatment group and the basal group.

#### 2.5.3. Biochemical Serum Parameters in Wistar Rats Treated with *Baccharis dracunculifolia* DC Extract

**Figure 5:**
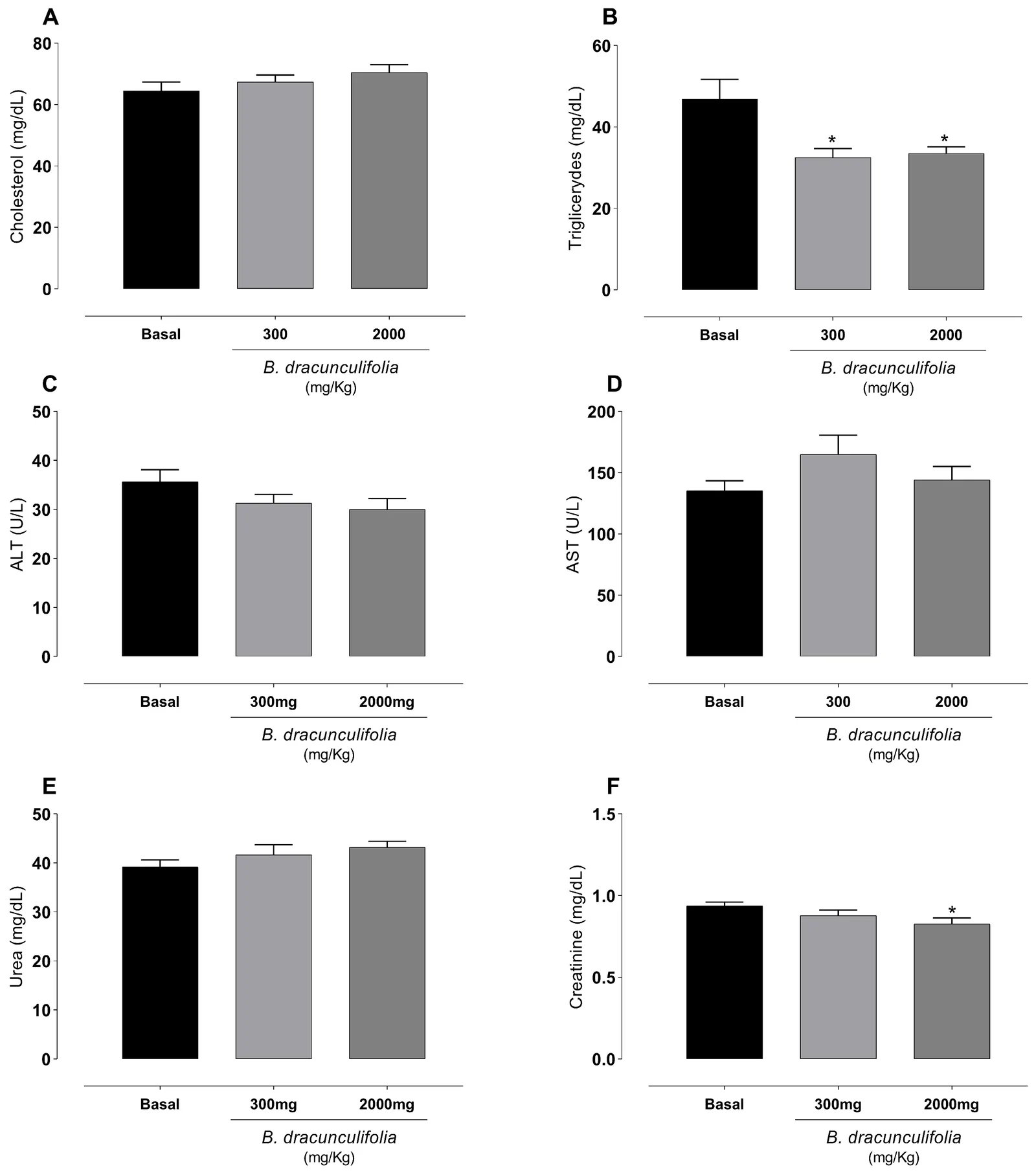
Serum biochemical parameters of (**A**) cholesterol, (**B**) triglycerides, (**C**) ALT, (**D**) AST, (**E**) Urea and (**F**) creatinine of Wistar rats treated with vehicle (filtered water) or *Baccharis dracunculifolia* extract (300 or 2000 mg/kg) via oral administration as a single dose. Statistical analysis was performed using one-way ANOVA followed by Tukey’s post hoc test. * *p* < 0.05 compared to the basal group. AST: aspartate aminotransferase; ALT: alanine aminotransferase.

The biochemical analysis of serum parameters in Wistar rats treated with *B. dracunculifolia* extract revealed significant changes in specific parameters (Figure 5). Triglyceride levels were significantly lower in both treatment groups than in the vehicle control group, with values of 46.83 ± 4.84 mg/dL in the control group, 32.50 ± 2.17 mg/dL in the 300 mg/kg group, and 33.50 ± 1.62 mg/dL in the 2000 mg/kg group (Figure 5B). Creatinine levels were also significantly lower in the 2000 mg/kg group, with values of 0.93 ± 0.02 mg/dL, 0.87 ± 0.03 mg/dL, and 0.82 ± 0.03 mg/dL in the control, 300 mg/kg, and 2000 mg/kg groups, respectively (Figure 5F). No statistically significant differences were observed for the other biochemical parameters analyzed. Cholesterol levels showed a slight, non-significant increase, with a value of 64.50 ± 2.88 mg/dL in the control group (Figure 5A). Urea levels remained stable across groups, with a control value of 39.17 ± 1.47 mg/dL (Figure 5E). Similarly, the liver enzymes aspartate aminotransferase (AST) and alanine aminotransferase (ALT) showed no significant differences among groups, with control values of 135.50 ± 7.90 U/L and 35.67 ± 2.47 U/L, respectively (Figure 5C,D).

#### 2.5.4. Hematological Profile of Rats Treated with *Baccharis dracunculifolia* DC Extract

Regarding the hematological parameters, no significant differences were observed among the groups in erythrocyte count, hemoglobin, hematocrit, mean corpuscular volume (MCV), mean corpuscular hemoglobin (MCH), or mean corpuscular hemoglobin concentration (MCHC). Similarly, no significant differences were observed in total leukocyte counts or in leukocyte subtypes, including segmented neutrophils, lymphocytes, and monocytes. Platelet counts also showed no significant differences among the groups (Table 8).

#### 2.5.5. *Baccharis dracunculifolia* DC Does Not Induce Hepatic, Cardiac and Renal Cellular Alterations

**Figure 6:**
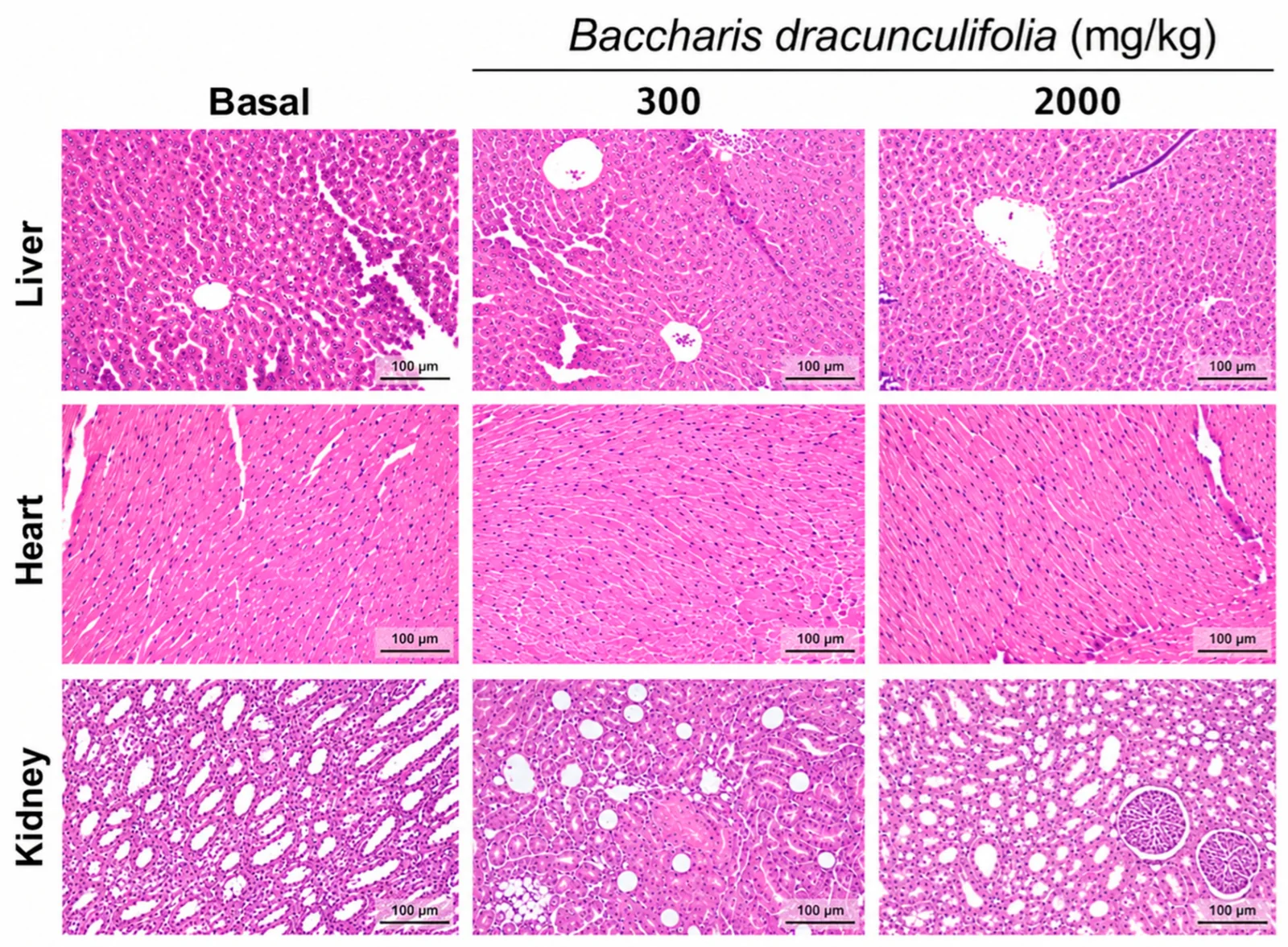
Histopathological analysis of the liver, heart, and kidney from Wistar rats treated with *Baccharis dracunculifolia* extract (300 mg/kg and 2000 mg/kg), compared to the basal (vehicle) group. Representative Hematoxylin & Eosin-stained tissue sections show no significant morphological alterations in the liver, heart, or kidney at both dosages of the extract. Magnification: 20×.

Histological examination of the liver, heart, and kidneys of Wistar rats treated with *B. dracunculifolia* extract at 300 and 2000 mg/kg revealed no treatment-related pathological alterations. Liver architecture remained preserved, with no evidence of necrosis, inflammation, or degeneration in either treatment group. Similarly, the heart maintained normal myocardial architecture, with no signs of tissue damage or inflammatory infiltration. The kidneys also exhibited preserved renal tubules and glomeruli, without evidence of interstitial inflammation or glomerular injury (Figure 6).

**Figure 7:**
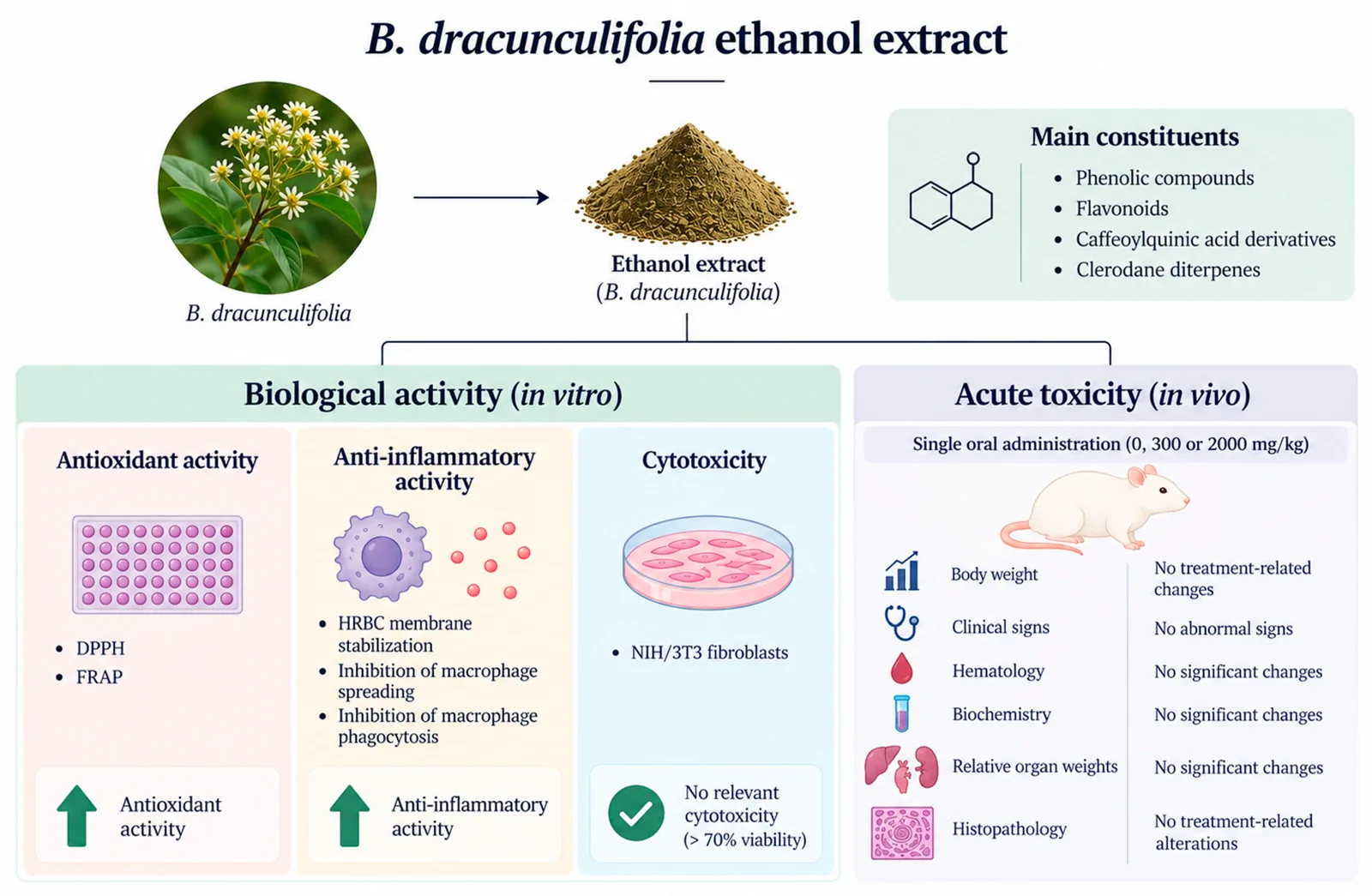
Integrated overview of the biological activity and acute toxicity profile of the *Baccharis dracunculifolia* DC. ethanol extract. The extract exhibited antioxidant activity in DPPH and FRAP assays, anti-inflammatory activity through HRBC membrane stabilization and inhibition of macrophage spreading and phagocytosis, and no relevant cytotoxicity in NIH/3T3 fibroblasts. In the acute oral toxicity assessment, no treatment-related adverse effects were observed in body weight, clinical signs, hematological or biochemical parameters, relative organ weights, or histopathological findings following single oral administration at 300 and 2000 mg/kg.

Overall, the biological and toxicological evaluation revealed a consistent profile for the *Baccharis dracunculifolia* DC. ethanol extract (Figure 7). The extract exhibited antioxidant activity in both DPPH and FRAP assays and concentration-dependent anti-inflammatory effects in the membrane stabilization, macrophage spreading, and phagocytosis assays. In parallel, the extract did not show relevant cytotoxicity toward NIH/3T3 fibroblasts at the tested concentrations. The acute oral toxicity assessment further demonstrated the absence of mortality or treatment-related alterations in clinical signs, body weight, hematological and biochemical parameters, relative organ weights, and histopathological findings at doses up to 2000 mg/kg. Collectively, these findings indicate a biological activity profile accompanied by a favorable acute toxicological profile under the experimental conditions evaluated.

## 3. Discussion

This study provides an integrated evaluation of the phytochemical composition, biological activities, and acute toxicological profile of *Baccharis dracunculifolia* DC. extract by combining untargeted UHPLC-QTOF-MS analysis with complementary in vitro and in vivo approaches. This strategy enabled the characterization of the chemical diversity of the extract and the assessment of its antioxidant and anti-inflammatory activities, cytotoxicity, and acute oral toxicity. The absence of mortality, treatment-related behavioral changes, and histopathological alterations following acute oral exposure, together with the absence of relevant changes in hematological and biochemical parameters and relative organ weights, indicates a favorable acute toxicity profile under the tested conditions. The absence of treatment-related adverse effects at the highest dose tested (2000 mg/kg) is consistent with the classification framework of Organisation for Economic Co-operation and Development (OECD) Guideline 423 and supports the need for further toxicological investigations to characterize the safety of the extract under repeated-exposure conditions.

The UHPLC-QTOF-MS analysis revealed a chemically diverse profile, with the tentative annotation of 54 metabolites belonging to several phytochemical classes, including phenolic acids, flavonoids, caffeoylquinic acid derivatives, diterpenes, glycolipids, saponins, terpenoids, and other specialized metabolites. Among the tentatively annotated compounds, chlorogenic acid isomers, dicaffeoylquinic acids, vicenin derivatives, quercetin glycosides, kaempferide, Artepillin C, and clerodane diterpenes are of particular interest because of their previously reported biological activities [14,34]. The coexistence of these metabolites highlights the chemical complexity of the extract and suggests that its biological effects may result from the combined activity of multiple constituents rather than from a single compound.

The quantification of total phenolic and flavonoid contents provided additional information on the response of the crude *B. dracunculifolia* extract at different sample concentrations. The measured total phenolic content increased with increasing extract concentration, which is expected because higher concentrations of the extract provide a greater amount of analyte in the reaction system. Therefore, these results should not be interpreted as evidence of enrichment of the crude extract in phenolic compounds. The total flavonoid content showed a less pronounced and non-linear variation among the tested concentrations, with the highest value observed at 0.25 mg/mL and a decrease at 0.75 mg/mL, followed by a slight increase at 1.00 mg/mL. These variations may reflect differences in the analytical response at the concentrations evaluated and should therefore be interpreted cautiously.

Previous phytochemical investigations of *B. dracunculifolia* have reported the presence of phenolic compounds and flavonoids, supporting the occurrence of these classes of secondary metabolites in the species [24,37,38,39,40]. The metabolomic profile obtained in the present study further corroborates the chemical diversity of the extract by demonstrating the simultaneous occurrence of compounds belonging to different biosynthetic pathways, including hydroxycinnamic acids, flavonoid glycosides, diterpenes, glycolipids, and terpenoids. This chemical diversity may contribute to the biological properties of the extract, although the present results do not allow specific synergistic or additive interactions among individual constituents to be established. From a pharmacological perspective, the presence of multiple classes of compounds highlights the potential for interactions among constituents with different molecular targets. However, further mechanistic studies are required to confirm such interactions and to determine their contribution to the biological effects observed.

The antioxidant activity demonstrated in the DPPH and FRAP assays indicates that the extract can act as both a radical scavenger and an electron donor, in agreement with previous reports [14,15,16]. These effects may be associated, at least in part, with the phenolic compounds tentatively annotated by UHPLC-QTOF-MS. The presence of chlorogenic acid derivatives, dicaffeoylquinic acids, flavonoid glycosides, including quercetin and vicenin derivatives, as well as kaempferide, Artepillin C, and clerodane diterpenes, provides a plausible chemical basis for the antioxidant activity observed. These compounds have been individually associated with complementary antioxidant mechanisms, including free radical scavenging, hydrogen atom donation, metal ion chelation, modulation of intracellular redox signaling, activation of the Nrf2 antioxidant pathway, and inhibition of oxidative damage [41,42,43,44]. Rather than attributing the observed antioxidant activity to a single constituent, the combined presence of multiple phytochemical classes may contribute to the overall antioxidant effect of *B. dracunculifolia* extract.

Although the antioxidant activity observed was lower than that of the reference compounds, the extract still demonstrated measurable antioxidant capacity under the experimental conditions. Rather than acting exclusively as direct radical scavengers, plant-derived phytochemicals are increasingly recognized as modulators of cellular redox homeostasis, potentially enhancing endogenous antioxidant defenses while preserving the physiological functions of reactive oxygen species in cell signaling [45,46]. This concept is particularly relevant because excessive antioxidant activity may produce paradoxical pro-oxidant effects under certain experimental conditions. Therefore, the antioxidant capacity observed in the present study may reflect the combined contribution of multiple phytochemical classes rather than the activity of a single metabolite.

The in vitro anti-inflammatory assays revealed a clear concentration-dependent effect, with greater inhibition of hemolysis, cell spreading, and phagocytosis at higher extract concentrations. These findings indicate membrane-stabilizing and immunomodulatory effects under the experimental conditions evaluated. Membrane stabilization may be related to interactions between phenolic compounds and phospholipid bilayers, which may reduce membrane fragility and protect erythrocytes from lysis. In addition, the inhibition of macrophage spreading and phagocytosis indicates modulation of macrophage activity and may reflect effects on inflammatory signaling pathways. Although these mechanisms were not directly investigated in the present study, previous studies of *Baccharis* species have described effects involving pathways such as NF-κB and the regulation of pro-inflammatory mediators [47,48,49,50].

The phytochemical profile obtained by UHPLC-QTOF-MS provides a plausible chemical basis for these findings. Chlorogenic acid and dicaffeoylquinic acid derivatives have been reported to attenuate inflammatory responses by suppressing NF-κB activation, reducing the production of nitric oxide, prostaglandins, and pro-inflammatory cytokines, and modulating intracellular oxidative stress [41]. Likewise, flavonoid derivatives tentatively annotated in the extract, including vicenin glycosides, quercetin glycosides, and kaempferide, have been associated with inhibition of inflammatory enzymes, reduction of reactive oxygen species, and modulation of macrophage activation [51,52,53,54]. The tentative annotation of Artepillin C is also consistent with previous reports describing this prenylated phenolic compound as a modulator of inflammatory signaling pathways, including NF-κB and MAPK, and as a potential suppressor of cytokine production [43]. However, these mechanisms were not directly investigated in the present study, and the contribution of individual constituents to the observed anti-inflammatory activity cannot be established from the current data.

In addition to phenolic constituents, the tentative annotation of several clerodane diterpenes may also contribute to the anti-inflammatory activity observed. Clerodane diterpenes are characteristic metabolites of several Baccharis species and have been reported to exhibit anti-inflammatory, antioxidant, antimicrobial, and immunomodulatory activities through multiple molecular targets [55]. Although the individual contribution of each metabolite cannot be established in the present study, the coexistence of these compounds supports the hypothesis that the biological activity of *B. dracunculifolia* may involve the combined contribution of multiple phytochemical classes acting through complementary mechanisms. However, it is important to consider that excessive suppression of phagocytic activity could potentially compromise host defense mechanisms, reinforcing the importance of dose optimization in future pharmacological studies.

The anti-inflammatory activity observed in the present study may be relevant to pathological conditions in which excessive inflammatory signaling and oxidative imbalance contribute to tissue injury. Previous preclinical studies have provided disease-oriented evidence supporting the biological potential of *B. dracunculifolia*. Hydroalcoholic extracts of the species have demonstrated antiulcer activity in experimental models of gastric injury [56] and promoted healing of established gastric ulcers [56]. Similarly, *B. dracunculifolia* extracts attenuated intestinal damage and inflammatory responses in an experimental model of colitis [57], while its essential oil exhibited anti-inflammatory effects in models of skin inflammation [47]. Together, these findings provide a disease-oriented context for the anti-inflammatory activity observed in the present study and suggest that *B. dracunculifolia* may be of interest for further investigation in inflammatory gastrointestinal and cutaneous conditions. However, these effects are supported primarily by preclinical evidence and cannot be directly extrapolated to clinical efficacy. Further studies are therefore needed to determine the underlying mechanisms, active constituents, pharmacological relevance, and safety of *B. dracunculifolia* preparations in disease-specific models.

The biochemical profile further supports the absence of evidence of hepatic or renal toxicity under the experimental conditions, as no toxicologically relevant alterations were observed in classical biochemical markers of hepatic and renal function, including AST, ALT, and urea levels. Interestingly, the reduction in triglyceride levels observed in the present study may also be relevant in light of previous in vivo evidence from our group. In an experimental model of cardiometabolic syndrome characterized by diabetes, dyslipidemia, and cigarette smoke exposure, *B. dracunculifolia* extract reduced both plasma and hepatic lipid levels, including triglycerides and cholesterol, and attenuated hepatic steatosis [24]. The consistency between these findings and the triglyceride reduction observed here suggests that the lipid-modulatory effects of *B. dracunculifolia* warrant further investigation in disease-oriented cardiometabolic models. Importantly, however, the present study was not designed to establish a therapeutic effect on dyslipidemia; therefore, the isolated reduction in triglycerides should be interpreted as a potential pharmacological signal rather than evidence of therapeutic efficacy. Moreover, the observed biochemical changes were not accompanied by alterations in other evaluated indicators of hepatic or renal injury, supporting further investigation into whether the triglyceride-lowering effect represents a reproducible metabolic action of the extract or a transient response to acute exposure.

The potential biological basis of the lipid-modulatory effects of *B. dracunculifolia* may be associated not only with its phenolic constituents but also with caffeoylquinic acid derivatives and flavonoids, which have been reported to influence lipid synthesis, fatty acid oxidation, and hepatic lipid accumulation [42]. Similarly, Artepillin C has been investigated for its potential effects on metabolic homeostasis, including modulation of lipid metabolism and insulin sensitivity [43]. Although these findings should be interpreted with caution, they provide a plausible context for the triglyceride reduction observed in the present study. However, because the mechanisms underlying this effect were not directly investigated, the contribution of individual phytochemical constituents cannot be established. Further studies using disease-oriented models and mechanistic approaches are needed to determine whether *B. dracunculifolia* exerts reproducible lipid-modulatory effects.

Likewise, the slight reduction in creatinine levels does not indicate renal injury and may be consistent with preserved renal function under the experimental conditions. Considering the absence of histopathological alterations and the maintenance of urea concentrations, these findings collectively support the absence of evidence of renal toxicity under the conditions evaluated. Nevertheless, the present study was not designed to investigate renal pharmacological effects, and dedicated studies are required to determine whether the identified metabolites exert any direct effects on renal function or whether the observed changes simply reflect normal biological variation.

Hematological parameters remained within the expected physiological ranges, with no significant changes in erythrocyte, platelet, or leukocyte parameters, indicating that acute exposure to the extract did not produce detectable alterations in hematological profiles [58,59,60]. Similarly, the normal progression of body weight gain and the absence of significant changes in relative organ weights further support the absence of systemic toxicity under the experimental conditions. Histopathological analysis corroborated these findings, revealing preserved tissue architecture in the liver, heart, and kidneys, with no evidence of inflammation, necrosis, fibrosis, or degenerative changes. Taken together, the absence of relevant hematological, gross morphological, and histopathological alterations supports the favorable acute toxicity profile of the extract following oral exposure.

Importantly, the complementary nature of the in vitro and in vivo findings provides a broader characterization of the biological and acute toxicological profiles of *B. dracunculifolia* extract. While the in vitro assays demonstrated antioxidant and anti-inflammatory activities without relevant cytotoxicity under the tested conditions, the in vivo evaluation showed no evidence of treatment-related systemic toxicity following acute oral exposure. This convergence of findings highlights the value of integrating complementary experimental approaches in the preclinical characterization of medicinal plants. In addition, the comprehensive UHPLC-QTOF-MS metabolite profiling provides a chemical framework for interpreting the observed biological responses, allowing the biological findings to be considered in the context of the chemically diverse composition of the extract [61]. Such integrated approaches may contribute to the characterization and future standardization of botanical preparations intended for pharmaceutical applications, although further pharmacological, toxicological, and chemical studies are required to establish the contribution of individual constituents to the observed effects [62].

From a toxicological standpoint, the absence of treatment-related adverse effects at doses up to 2000 mg/kg indicates a favorable acute toxicity profile under the conditions evaluated. However, extrapolation of these findings to human use requires caution, as interspecies differences in metabolism, exposure duration, formulation, and bioavailability may influence toxicological outcomes. Furthermore, the biological activity of botanical extracts depends not only on the presence of individual metabolites but also on their relative abundance, chemical interactions, and metabolic transformation following ingestion. These factors reinforce the importance of comprehensive and reproducible phytochemical characterization in future pharmacological and toxicological investigations [63].

An important consideration is the variability in toxicological findings reported in the literature. While the present study demonstrated a favorable acute toxicity profile under the conditions evaluated, previous reports have described genotoxic or mutagenic effects associated with *Baccharis*-derived compounds [25,64,65,66]. These discrepancies may reflect differences in extraction methods, geographical origin of the plant material, seasonal variation, chemical composition, experimental models, and exposure protocols. Such variability further emphasizes the importance of comprehensive phytochemical characterization, as differences in metabolite composition may influence both biological activity and toxicological outcomes. In this context, integrating chemical profiling with standardized and reproducible biological assays may improve the comparability and reproducibility of studies involving medicinal plants. In addition, complementary genotoxicity assays, such as the comet assay and micronucleus test, could provide further information for the toxicological characterization of *B. dracunculifolia* extracts in future studies.

Despite the promising findings, some limitations should be acknowledged. Although the UHPLC-QTOF-MS analysis provided a comprehensive overview of the metabolite composition, compound identification was based on tentative annotation from accurate mass measurements and fragmentation patterns. Therefore, confirmation using authentic reference standards and quantitative targeted analyses would provide additional confidence regarding the identity and abundance of the detected metabolites [67]. Moreover, the in vitro assays employed do not fully reproduce the complexity of mammalian physiological systems, and the present study evaluated only acute oral toxicity. The stability of the extract under physiological conditions was also not investigated. Consequently, further investigations addressing subchronic and chronic toxicity, pharmacokinetics, bioavailability, metabolism, stability under physiologically relevant conditions, and mechanisms of action are necessary. Future studies should also investigate the interactions between the identified metabolites and the gut microbiota, as well as possible synergistic or antagonistic effects among the major phytochemical classes identified in the extract.

## 4. Materials and Methods

### 4.1. Preparation of Baccharis dracunculifolia Extract

The aerial parts of *Baccharis dracunculifolia* DC. were collected in the municipality of Maria Helena, Paraná, Brazil (23°39′49.7″ S and 53°18′18.0″ W), and was registered in the Brazilian National System for Management of Genetic Heritage and Associated Traditional Knowledge (SisGen, nº A1F489A). The plant material was dried on plant drying mats at room temperature (32 °C) and fragmented using an industrial blender (SPOLU, model SPL-023-eco, Itajobi, Brazil). The resulting plant material was subjected to granulometric classification using sieves with nominal apertures of 1700, 850, and 710 µm. The powder was sequentially passed through the sieves, and the fraction retained on the 710 µm sieve was collected and used for the extraction procedure. The powdered plant material (230 g) underwent dynamic maceration using 96 °GL cereal alcohol (ethanol; Labsynth, Dianema, São Paulo, Brazil) as the extraction solvent, without additional dilution with water [68]. For each extraction cycle, 500 mL of ethanol was used, corresponding to a plant material-to-solvent ratio of approximately 1:2.17 (g/mL). The extraction was performed under orbital agitation at 20 rpm using an orbital shaker (Nova Ética, Vargem Grande Paulista, São Paulo, Brazil), with renewal of the extraction solvent every 2 days. A total of 20 extraction cycles were performed, using fresh 96 °GL ethanol (500 mL) for each cycle, until exhaustion of the plant material. The resulting extracts were filtered and concentrated under reduced pressure using a rotary evaporator (Tecnal^®^, model TE-211, Piracicaba, Brazil) at 40 °C until the crude extract was obtained. The final mass of the crude extract was 70.0 g, corresponding to an extraction yield of 30.46%. The extract was stored at −20 °C until further analysis.

### 4.2. Phytochemical Characterization of Baccharis dracunculifolia DC Extract

#### 4.2.1. UHPLC-QTOF-MS Analysis

The phytochemical analysis of *B. dracunculifolia* extract was performed in an LCMS-9050 UHPLC/Q-TOF system (Shimadzu, Kyoto, Japan). The column used was a Luna Omega C18 100 Å (100 × 2.1 mm; 1.6 µm particle size) (Phenomenex, Torrance, CA, USA), held in a column oven at 40 °C. The mobile phases were ultrapure water with 0.1% (*v*/*v*) formic acid LC/MS grade (A) and acetonitrile with 0.1% (*v*/*v*) formic acid LC/MS grade (B). The analysis were performed using the following gradient: 0–5 min, 2–5% of B; 5–20 min, 5–30% of B; 20–30 min, 30–80% of B; 30–35 min, 80% of B; 35–36 min, 80–100% of B; 36–39 min 100% of B, 39–40 min 100-2% of B; 40–45% min 2% of B, with a total running time of 45 min. The flow rate was maintained at 0.3 mL/min, the injection volume was 1 µL, and the sample was held at 4 °C in the autosampler during analyses.

The sample was prepared at a concentration of 1 mg/mL in methanol–water (1:1, *v*/*v*) and analyzed in both positive and negative ionization modes. The MS interface was configured with a nebulizing gas flow rate of 3 L/min, heating and drying gas flow rates of 10 L/min, an interface temperature of 300 °C, a desolvation line temperature of 250 °C, a heat block temperature of 400 °C, and an interface voltage of 3 kV in positive mode and −3 kV in negative mode. For mass spectrometric data acquisition, a data-dependent analysis (DDA) method was used in both positive and negative ionization modes, with five dependent events, a start delay time of 0.8 s, and a total loop time of 0.8 s. The TOF-MS range was set to *m*/*z* 100–2000, while the TOF-MS/MS range was set to *m*/*z* 50–1500. Collision energy was applied in spread mode over a range of 20–40 eV, using argon at 270 kPa as the collision gas.

The data analysis was performed on the LabSolutions Insight Explorer CSD v. 1.00 software (Shimadzu, Kyoto, Japan). The structural annotations were performed based on MS/MS spectra from the experimental analysis, and spectral comparisons were performed using the PubChem (https://pubchem.ncbi.nlm.nih.gov), MoNA (https://mona.fiehnlab.ucdavis.edu), and Lotus (https://lotus.naturalproducts.net) databases, accessed between June and July 2026, as well as available literature reports.

#### 4.2.2. Monosaccharide Analysis

For monosaccharide analysis, we adapted the sample preparation from [31] as follows: the extract (1 mg) was hydrolyzed in 1.5 M trifluoroacetic acid at 100 °C for 5 h to release monosaccharides. The acid solution was evaporated under an N2 stream, resuspended in water, and reduced with 5 mg NaBH4 at room temperature for 15 h. Cationic resin was used to remove excess sodium, and residual borate was eliminated by successive co-evaporation in methanol under low pressure. The resulting alditols were acetylated with 1 mL of acetic anhydride/pyridine (1:1, *v*/*v*) for 1 h at 100 °C. The resulting alditol acetates were extracted in chloroform, and the excess of reagents was removed by successive water partitioning. After this cleanup, the chloroform was evaporated under an N2 stream.

The analysis was performed by gas chromatography-mass spectrometry in a GC-2010 Plus coupled with a QP-2020 single quadrupole mass spectrometer (Shimadzu Co., Ltd., Kyoto, Japan). The column used was an Rtx-5MS, 30 m x 0.25 mm (L × D), 0.25 µm film thickness (Shimadzu Co., Ltd., Kyoto, Japan). The temperature set was as follows: initial at 50 °C, hold for 1 min, then increased to 150 °C at a rate of 20 °C/min, then increased to 200 °C with 5 °C/min, hold for 5 min, and then increased to 250 °C at 25 °C/min, hold for 5 min. Total running time was 28 min. The sample was dissolved in 1 mL acetone, and the injection volume was 1 µL in split mode (1:10 ratio). Injection temperature was 250 °C, and helium 5.0 (analytical grade) was used as the carrier gas with a flow of 1.54 mL/min. The MS parameters were as follows: electron ionization source at 70 eV, temperature of 250 °C, interface temperature at 250 °C, acquisition in scan mode, ranging from 50–500 *m*/*z*, with an event time of 0.3 s and a scan time from 3.5 to 28 min.

#### 4.2.3. Total Flavonoid Content

The total flavonoid content was determined using the colorimetric method described by Alves and Kubota [69], with quercetin used as the reference standard. The *B. dracunculifolia* extract was diluted in methanol–water (80:20, *v*/*v*) to prepare a stock solution at a concentration of 1 mg/mL. Three additional dilutions were prepared from the stock solution at concentrations of 0.25, 0.50, and 0.75 mg/mL. For each dilution, an aliquot of 0.5 mL was mixed with 0.5 mL of 2% aluminum chloride solution (*w*/*v*) and incubated at room temperature for 10 min. The absorbance was measured at 425 nm using a UV–Vis spectrophotometer (Kasuaki, model IL-593-BI, Wuxi, China). Flavonoid concentration was calculated using a quercetin standard curve (5–40 µg/mL), and the aluminum chloride solution was used as the analytical control. The results were expressed as micrograms of quercetin equivalents per milligram of sample (μg QE/mg).

### 4.3. Antioxidant Studies

#### 4.3.1. Determination of Antioxidant Activity by DPPH Radical Scavenging Assay

The 2,2-diphenyl-1-picrylhydrazyl (DPPH) radical scavenging activity was evaluated according to Rufino et al. [70]. The *B. dracunculifolia* extract was diluted in methanol to obtain concentrations of 0.25, 0.50, 0.75, and 1.00 mg/mL. An aliquot of 0.1 mL of each dilution was added to 3.9 mL of a methanolic DPPH solution (60 μM). The reaction mixtures were incubated in the dark for 30 min at room temperature, and absorbance was measured at 515 nm using a UV–Vis spectrophotometer. A DPPH solution without the extract was used as the negative control, while quercetin (60 μM) was used as the reference antioxidant. The radical scavenging activity was calculated as follows:

Radical scavenging activity (%) = [(A_0_ − A_s_)/A_0_] × 100

where A_0_ is the absorbance of the control and A_s_ is the absorbance of the sample. The IC_50_ value, defined as the concentration required to inhibit 50% of DPPH radical activity, was determined by plotting radical scavenging activity against extract concentration.

#### 4.3.2. Determination of Antioxidant Activity by Ferric Reducing Antioxidant Power (FRAP) Assay

The FRAP assay was conducted according to the method described by Rufino et al. [71]. The *B. dracunculifolia* extract was diluted in methanol to obtain concentrations of 0.25, 0.50, 0.75, and 1.00 mg/mL. An aliquot of 90 μL from each dilution was mixed with 270 μL of distilled water and 2.7 mL of FRAP reagent [composed of 25 mL of acetate buffer (0.3 M), 2.5 mL of an aqueous solution of 2,4,6-tris(2-pyridyl)-*s*-triazine (TPTZ, 10 mM), and 2.5 mL of an aqueous solution of ferric chloride (20 mM)]. The mixtures were incubated at 37 °C for 30 min, and absorbance was measured at 595 nm using a UV–Vis spectrophotometer (Kasuaki, model IL-593-BI, Wuxi, China). A ferric sulfate standard curve (0–1000 μM) was used to express the antioxidant activity as micromoles of ferric sulfate equivalents per milligram of sample (μM FeSO_4_/mg).

### 4.4. In Vitro Potential Anti-Inflammatory Activity of Baccharis dracunculifolia

#### 4.4.1. Inhibition of Hemolysis

The human red blood cell (HRBC) membrane stabilization assay was performed according to Ananthi and Chitra [72]. The reaction mixture consisted of 2 mL of hyposaline solution (0.18%), 1 mL of sodium phosphate buffer (0.1 M, pH 7.4), and 1 mL of *B. dracunculifolia* extract at concentrations of 200, 400, and 600 μg/mL. Dexamethasone (40 μg/mL) was used as the positive control (PC), while 0.9% saline solution was used as the negative control (NC). Subsequently, 0.5 mL of HRBC suspension was added to each reaction mixture.

The samples were incubated under the conditions specified in the referenced method, followed by centrifugation. The hemoglobin content in the supernatant was then measured spectrophotometrically at 560 nm. The percentage of protection against hemolysis was calculated using the following equation:

Protection (%) = [(E_0_ − E_t_)/E_0_] × 100

where E_0_ represents the mean absorbance of the negative control group and Eₜ represents the mean absorbance of the treated groups.

#### 4.4.2. Treatments of Macrophages Cell

The murine macrophage cell line RAW 264.7 was used for both the macrophage spreading and phagocytosis inhibition assays. The crude *B. dracunculifolia* extract was dissolved in distilled water at concentrations of 200, 400, and 600 μg/mL. Dexamethasone (40 μg/mL) was used as the PC, whereas the NC consisted of a 0.9% saline solution [73,74].

#### 4.4.3. Inhibition of Spreading

The inhibition of cell spreading was evaluated according to the method described by Bastos et al. [74]. Aliquots (100 μL) of peritoneal lavage cell suspension, adjusted to 5.0 × 10^5^ cells/mL, were cultured in high-glucose DMEM supplemented with 10% fetal bovine serum and 1% penicillin–streptomycin–amphotericin. The cell suspensions were placed onto glass slides and allowed to adhere for 15 min at 25 °C. Subsequently, non-adherent cells were removed by washing with sterile saline solution. The adherent cells were then incubated for 1 h at 37 °C with 100 μL of DMEM and 10 μL of *B. dracunculifolia* extract at concentrations of 200, 400, and 600 μg/mL. Dexamethasone (0.1 mg/mL) was used as the PC, while 0.9% saline solution was used as the NC. After incubation, the slides were washed with sterile saline solution, and the adherent cells were fixed with 2.5% glutaraldehyde for 5 min. The cells were stained with Wright’s stain and mounted using Entellan. The slides were examined under an optical microscope at 400× magnification, and 100 cells were counted per slide. All experiments were performed in triplicate. The percentage of spreading inhibition was calculated using the following equation:

Inhibition of spreading (%) = [(E_0_ − E_t_)/E_0_] × 100

where E_0_ represents the mean number of spread cells in the negative control group and Eₜ represents the mean number of spread cells in the treated groups.

#### 4.4.4. Phagocytosis

The phagocytosis inhibition assay was performed according to Azedo et al. [73]. Briefly, 200 μL of macrophage suspension (3 × 10^5^ cells/mL) were seeded into 24-well plates containing glass coverslips and allowed to adhere overnight. After adhesion, the cells were washed with phosphate-buffered saline to remove non-adherent cells. The adherent cells were then incubated for 2 h in culture medium containing *B. dracunculifolia* extract at concentrations of 200, 400, and 600 μg/mL. Dexamethasone (0.1 mg/mL) was used as the PC, whereas 0.9% saline solution was used as the NC. The plates were maintained in a humidified incubator at 37 °C with 5% CO_2_. Following incubation, the cells were washed three times with phosphate-buffered saline, fixed with 2.5% glutaraldehyde, and stained with Wright’s stain. The coverslips were mounted onto permanent slides for analysis. The slides were examined under an optical microscope at 400× magnification, and 100 cells were counted per sample. All experiments were performed in triplicate. The percentage of phagocytosis inhibition was calculated using the following equation:

IP (%) = [(E_0_ − Eₜ)/E_0_] × 100

where E_0_ represents the mean number of cells in the negative control group that phagocytosed zymosan particles, and Eₜ represents the mean number of cells in the treated groups.

### 4.5. Toxicological Studies

#### 4.5.1. In Vitro Cytotoxicity of *Baccharis dracunculifolia*

The cytotoxicity of *B. dracunculifolia* extract was evaluated using the MTT assay, according to Tsuboy et al. [75]. NIH/3T3 mouse dermal fibroblasts (ATCC^®^ CRL-1658™) were seeded into 96-well plates and cultured for 24 h at 37 °C in a humidified atmosphere containing 5% CO_2_. After reaching approximately 70–80% confluence, the cells were treated with *B. dracunculifolia* extract at concentrations of 100, 200, 400, 800, and 1600 μg/mL. The NC consisted of cells treated with physiological saline solution, whereas the PC consisted of cells treated with 2% (*v*/*v*) Tween 80. Treatments were performed for 24, 48, and 72 h. Following treatment, MTT solution was added to each well and incubated under standard conditions to allow for formazan crystal formation. The resulting crystals were solubilized, and absorbance was measured at 570 nm using a microplate reader. Cell viability was expressed as a percentage relative to the negative control. Cytotoxicity was interpreted based on OECD guidelines [76,77], ISO 10993-5:2009 [77], and previous studies [78,79,80], considering the established cell viability thresholds for the classification of cytotoxic effects.

#### 4.5.2. Acute Toxicity Evaluation of Baccharis dracunculifolia

##### Animals

The acute oral toxicity of the *B. dracunculifolia* aerial-part extract was evaluated in female Wistar rats weighing 200–250 g, obtained from the central animal facility of the Federal University of Grande Dourados. The animals were housed at the Experimental Neuroscience Laboratory of Universidade Paranaense under controlled environmental conditions (temperature, 22 ± 2 °C; relative humidity, 50 ± 10%; 12 h light/dark cycle), with environmental enrichment and free access to food and water. The experimental protocol was approved by the Ethics Committee on Animal Experimentation of UNIPAR (protocol no. 1003/2022), and all procedures were conducted in accordance with the guidelines and recommendations of the Brazilian National Council for the Control of Animal Experimentation (CONCEA). The study also adhered to the principles of Animal Research: Reporting of In Vivo Experiments (ARRIVE) [81], with measures taken to ensure animal welfare and minimize the number of animals used.

##### Experimental Design

To assess acute oral toxicity, female rats were tested according to the sequential procedure described in OECD Guideline 423 [82]. In this procedure, animals are tested sequentially in sets of three, with the subsequent step determined according to the occurrence of mortality or signs of toxicity. In the present study, the initial dose was set at 300 mg/kg, as no previous data regarding the acute toxicity of *B. dracunculifolia* extract were available in the literature. After an 8 h fasting period, the *B. dracunculifolia* extract was administered orally (gavage) as a single dose. Following treatment, the animals remained fasted for an additional 4 h to prevent potential interactions.

**Figure 8:**
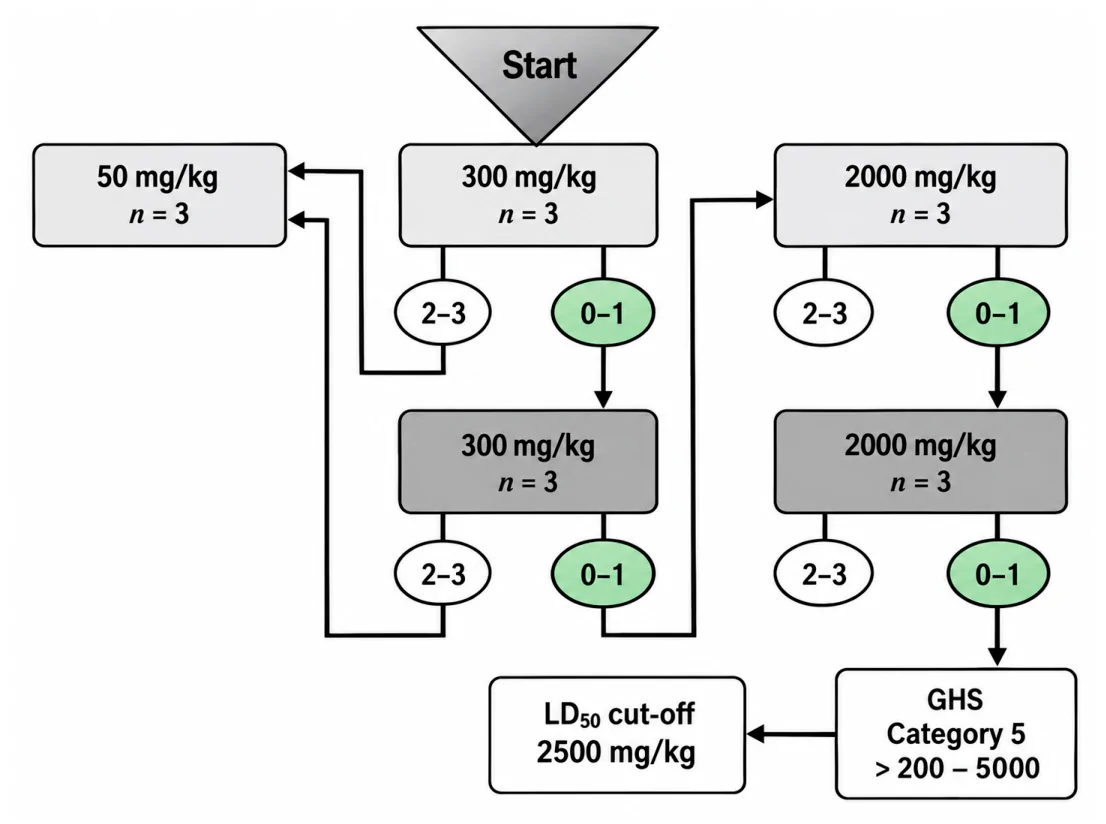
Acute toxicity test conducted following OECD Guideline 423, illustrating the stepwise process for determining the median lethal dose (LD_50_) cut-off value of *Baccharis dracunculifolia* DC. extract. This method involves sequential testing with three animals of the same sex per step. The acute toxicity assessment is based on the mortality or moribund state of the animals, requiring 2–4 steps to complete the evaluation. The initial dose (300 mg/kg in this study) is administered once. If 2–3 animals die, testing proceeds at a lower fixed dose. If one or no animals die, the initial dose is retested twice, followed by testing at a higher fixed dose in subsequent steps until the appropriate Globally Harmonized System toxicity category is identified. GHS: Globally Harmonized Classification System (mg/kg).

Three animals initially received 300 mg/kg. As no mortality or treatment-related signs of toxicity were observed, an additional three animals were subsequently administered the same dose, resulting in a total of six animals tested at 300 mg/kg. The procedure was then continued at 2000 mg/kg using the same sequential approach: three animals were initially treated, followed by an additional three animals because no mortality or relevant signs of toxicity were observed. Thus, a total of six animals were tested at 2000 mg/kg. Overall, 12 animals received the *B. dracunculifolia* extract, with three animals treated per sequential step. A control group (*n* = 6) was included as a baseline and received the vehicle (filtered water; Figure 8).

##### Assessment of Clinical Signs

Following administration of the *B. dracunculifolia* extract, the animals were initially observed at the following time points: 30 min, 1, 2, 3, and 4 h post-administration. Clinical signs, including self-grooming, piloerection, dyspnea, abdominal contractions, diarrhea, prostration, ataxia, sedation, coma, and death, were individually monitored and recorded. After the initial 4 h observation period, the animals were provided with food and water and continued to be monitored daily until day 14 for the occurrence of any clinical signs or death, in accordance with the study by Silva et al. [83].

##### Euthanasia and Material Collection

The animals were euthanized on day 15 by deepening anesthesia with isoflurane (1–3%) in an induction chamber. Following euthanasia, blood was collected by decapitation using a guillotine for hematological and biochemical analyses. The liver, spleen, kidneys, and heart were then carefully dissected and weighed using an analytical balance. Relative organ weight (%) was calculated by multiplying the weight of each organ by 100 and dividing it by the animal’s body weight. For the kidneys, the relative weight was calculated using the mean weight of both kidneys. Small samples of each organ were subsequently collected, washed with saline solution, and separated for histological processing.

##### Biochemical Analyses

The collected blood samples were centrifuged at 3000× *g* for 10 min, after which the plasma was stored at −80 °C. Biochemical analyses were performed using commercial kits and different analytical methods, including enzymatic-colorimetric methods for total cholesterol and triglycerides, kinetic-colorimetric methods for creatinine, kinetic UV methods for urea, and colorimetric methods for AST and ALT, using a semiautomatic analyzer (Global Analyzer^®^, model GTA-300, Global Trade Technology, Piracicaba, Brazil).

##### Hematological Analyses

The hematological profile was evaluated by analyzing the erythrocyte series (erythrocyte count, hemoglobin, hematocrit, MCV, MCH, and MCHC), leukocyte parameters (total leukocyte count and differential leukocyte count, including band and segmented neutrophils, lymphocytes, and monocytes), and platelet count using an automated hematological analyzer (ABX Micros ESV 60, Paris, France). The differential leukocyte count was performed using blood smears stained with Giemsa and examined under an optical microscope (Leica^®^, model DM 2500, Wetzlar, Germany).

##### Microscopic Analysis of Tissue Structure

Fragments of the liver, spleen, heart, and kidneys were fixed in a solution composed of 1800 mL of distilled water, 200 mL of formaldehyde, 8 g of monosodium phosphate, and 13 g of disodium phosphate (10% buffered formalin). After standard histological processing, the sections were stained with hematoxylin and eosin and analyzed histopathologically by a veterinary pathologist. The slides were examined under an optical microscope (Leica^®^, model DM 2500, Wetzlar, Germany) to detect cellular alterations resulting from the treatments, as described by Lima et al. [84] and Souza et al. [85].

### 4.6. Statistical Analysis

The data were initially assessed for homogeneity of variance and normality of distribution. ANOVA was performed, followed by Tukey’s post hoc test for multiple comparisons. Statistical significance was set at *p* < 0.05 (α = 0.05). Statistical analyses and graph generation were performed using GraphPad Prism^®^ version 11.0.

## 5. Conclusions

In conclusion, the ethanol extract of *B. dracunculifolia* DC. exhibited a chemically diverse phytochemical profile, together with antioxidant and anti-inflammatory activities and a favorable acute toxicity profile under the experimental conditions evaluated. The integration of UHPLC-QTOF-MS profiling with complementary biological and toxicological approaches provides a comprehensive framework for characterizing the chemical composition, biological activities, and acute toxicity profile of the extract. In light of the present findings and previous preclinical evidence, the biological potential of *B. dracunculifolia* in inflammatory and gastrointestinal conditions warrants further investigation. Similarly, the observed reduction in triglyceride levels and previous evidence from a cardiometabolic model support further investigation of its potential metabolic effects. Nevertheless, these findings remain preclinical, and further studies addressing the mechanisms of action, pharmacokinetics, bioavailability, long-term safety, genotoxicity, and reproducible phytochemical characterization are required before any therapeutic application can be established.

## Figures and Tables

**Table 1:** Total phenolics and flavonoids of the *Baccharis dracunculifolia* DC. extract.

*B. dracunculifolia* (mg/mL)	Total Phenols Content (µg GAE/mg CE)	Flavonoid Content (µg QE/mg CE)
0.25	29.88 ± 7.17 ^d^	404.83 ± 13.44 ^a^
0.50	71.88 ± 1.44 ^c^	397.12 ± 2.86 ^ab^
0.75	102.15 ± 1.54 ^b^	355.75 ± 2.17 ^c^
1.00	132.48 ± 2.13 ^a^	379.40 ± 1.74 ^b^

Values are the mean ± standard deviation of triplicate assays. Data were subjected to analysis of variance (ANOVA), and differences between means were assessed by Tukey’s test (*p* ≤ 0.05). Values in the same column followed by different letters are significantly different (*p* ≤ 0.05). CE: Crude Extract. GAE/mg CE: Gallic Acid Equivalents/mg of Crude Extract; QE/mg CE: Quercetin Equivalents/mg of Crude Extract.

**Table 2:** Antioxidant activity, assessed by the elimination of the DPPH radical and FRAP assays of the *Baccharis dracunculifolia* DC. extract.

Samples mg/mL	DPPH• IC_50_ (mg/mL)	FRAP (µM Ferrous Sulfate/mg)
*B. dracunculifolia*	0.771 ± 0.009 ^b^	1.244 ± 0.223 ^b^
Quercetin	0.010 ± 0.00 ^a^	-
Trolox	-	9.175 ± 0.001 ^a^

Values are the mean ± standard deviation of triplicate assays. Data were subjected to analysis of variance (ANOVA), and differences between means were assessed by Tukey’s test (*p* ≤ 0.05). Values in the same column followed by different letters are significantly different (*p* ≤ 0.05). Quercetin (0.0103 mg/mL) and Trolox (9.175 mg/mL) were used as positive controls in DPPH• and FRAP assays, respectively. Abbreviations: FRAP: ferric reducing antioxidant power; IC_50_: half-maximal inhibitory concentration; DPPH•: 2,2-diphenyl-1-picrylhydrazyl.

**Table 3:** Percentage of hemolysis inhibition promoted by *Baccharis dracunculifolia* DC. extract at concentrations of 200, 400, and 600 μg/mL, compared to the positive and negative control groups.

Treatments	Hemolysis Inhibition (%)
NC	00.00 ± 0.00
PC	98.96 ± 0.34 *
*B. dracunculifolia* 200	45.43 ± 0.48 *
*B. dracunculifolia* 400	51.14 ± 0.86 *
*B. dracunculifolia* 600	58.60 ± 0.23 *

The results were expressed in mean ± S.E.M. One-way ANOVA followed by Tukey’s post hoc test was used for statistical analysis. * *p* < 0.05 compared to NC. *B. dracunculifolia*: µg/mL. NC: negative control (0.9% physiological solution); PC: positive control (dexamethasone, 40 μg/mL).

**Table 4:** Percentage of inhibition of spreading induced by *Baccharis dracunculifolia* DC. extract, compared to the positive and negative control groups.

Treatments	Inhibition of Spreading (%)
NC	0.00 ± 0.00
PC	91.97 ± 2.12 *
*B. dracunculifolia* 200	52.41 ± 0.69 *
*B. dracunculifolia* 400	54.22 ± 0.40 *
*B. dracunculifolia* 600	60.84 ± 0.40 *

The results were expressed in mean ± S.E.M. One-way ANOVA followed by Tukey’s post hoc test was used for statistical analysis. * *p* < 0.05 compared to NC. *B. dracunculifolia*: µg/mL. NC: negative control (0.9% physiological solution); PC: positive control (dexamethasone, 40 μg/mL).

**Table 5:** Percentage of phagocytosis inhibition promoted by *Baccharis dracunculifolia* DC. extract compared to the positive and negative control groups.

Treatments	Phagocytosis Inhibition (%)
NC	0.00 ± 0.00
PC	70.43 ± 2.87 *
*B. dracunculifolia* 200	44.96 ± 2.57 *
*B. dracunculifolia* 400	52.83 ± 1.35 *
*B. dracunculifolia* 600	57.89 ± 2.96 *

The results were expressed in mean ± S.E.M. One-way ANOVA followed by Tukey’s post hoc test was used for statistical analysis. * *p* < 0.05 compared to NC. *B. dracunculifolia*: µg/mL. NC: negative control (0.9% physiological solution); PC: positive control (dexamethasone, 40 μg/mL).

**Table 6:** Percentage of cell viability promoted by *Baccharis dracunculifolia* DC. extract at concentrations of 100, 200, 400, 800, and 1600 µg/mL, compared to the positive and negative controls groups.

Treatment	24 h	48 h	72 h
NC	100.00 ± 0.00	100.00 ± 0.00	100.00 ± 0.00
PC	1.84 ± 0.14 *	1.84 ± 0.14 *	1.37 ± 0.06 *
*B. dracunculifolia* 100	104.98 ± 0.14 *	96.87 ± 0.09	92.07 ± 0.06
*B. dracunculifolia* 200	102.19 ± 0.02	96.90 ± 0.02	87.13 ± 0.02 *
*B. dracunculifolia* 400	101.19 ± 0.02	92.37 ± 0.03 *	85.57 ± 0.03 *
*B. dracunculifolia* 800	95.93 ± 0.02	87.80 ± 0.01 *	80.86 ± 0.01 *
*B. dracunculifolia* 1600	80.23 ± 0.11 *	81.69 ± 4.30 *	78.63 ± 1.31 *

The results were expressed in mean ± S.E.M. One-way ANOVA followed by Tukey’s post hoc test was used for statistical analysis. * *p* < 0.05 compared to NC. *B. dracunculifolia*: µg/mL. NC: negative control (0.9% physiological solution); PC: positive control (0.1% Tween 80).

**Table 7:** Assessment of clinical signs in Wistar rats treated with vehicle or *Baccharis dracunculifolia* DC. extract (300 or 2000 mg/kg), orally, in a single dose.

Clinical Sign	Time					
	30 min	1 h	2 h	3 h	4 h	14 days
Basal group						
Self-cleaning absence	0/6	0/6	0/6	0/6	0/6	0/6
Piloerection	0/6	0/6	0/6	0/6	0/6	0/6
Dyspnea	0/6	0/6	0/6	0/6	0/6	0/6
Abdominal constriction	0/6	0/6	0/6	0/6	0/6	0/6
Diarrhea	0/6	0/6	0/6	0/6	0/6	0/6
Prostration	0/6	0/6	0/6	0/6	0/6	0/6
Ataxia	0/6	0/6	0/6	0/6	0/6	0/6
Sedation	0/6	0/6	0/6	0/6	0/6	0/6
Coma	0/6	0/6	0/6	0/6	0/6	0/6
Death	0/6	0/6	0/6	0/6	0/6	0/6
*B. dracunculifolia* (300 mg/kg)						
Self-cleaning absence	0/6	0/6	0/6	0/6	0/6	0/6
Piloerection	0/6	0/6	0/6	0/6	0/6	0/6
Dyspnea	0/6	0/6	0/6	0/6	0/6	0/6
Abdominal constriction	0/6	0/6	0/6	0/6	0/6	0/6
Diarrhea	0/6	0/6	0/6	0/6	0/6	0/6
Prostration	0/6	0/6	0/6	0/6	0/6	0/6
Ataxia	0/6	0/6	0/6	0/6	0/6	0/6
Sedation	0/6	0/6	0/6	0/6	0/6	0/6
Coma	0/6	0/6	0/6	0/6	0/6	0/6
Death	0/6	0/6	0/6	0/6	0/6	0/6
*B. dracunculifolia* (2000 mg/kg)						
Self-cleaning absence	0/6	0/6	0/6	0/6	0/6	0/6
Piloerection	0/6	0/6	0/6	0/6	0/6	0/6
Dyspnea	0/6	0/6	0/6	0/6	0/6	0/6
Abdominal constriction	0/6	0/6	0/6	0/6	0/6	0/6
Diarrhea	0/6	0/6	0/6	0/6	0/6	0/6
Prostration	0/6	0/6	0/6	0/6	0/6	0/6
Ataxia	0/6	0/6	0/6	0/6	0/6	0/6
Sedation	0/6	0/6	0/6	0/6	0/6	0/6
Coma	0/6	0/6	0/6	0/6	0/6	0/6
Death	0/6	0/6	0/6	0/6	0/6	0/6

**Table 8:** Hematological parameters of Wistar rats treated with vehicle or *Baccharis dracunculifolia* DC. extract (300 or 2000 mg/kg), orally, single dose.

Parameter	Basal	Extract of *Baccharis dracunculifolia*	
		300 mg/kg	2000 mg/kg
Red blood cells (10^6^ mm/µL)	9.12 ± 0.21	9.20 ± 0.21	8.89 ± 0.28
Hemoglobin (g/dL)	17.47 ± 0.81	17.80 ± 0.45	17.80 ± 0.55
Hematocrit (%)	53.75 ± 1.50	53.73 ± 1.05	53.17 ± 1.73
MCV (fl)	58.80 ± 0.33	57.72 ± 0.64	59.68 ± 0.45
MCH (pg)	19.05 ± 0.54	19.08 ± 0.38	19.97 ± 0.21
MCHC (g/dL)	32.28 ± 0.79	33.08 ± 0.50	33.50 ± 0.38
Leukocyte (10^3^ mm/µL)	4.97 ± 0.56	4.50 ± 0.42	3.97 ± 0.22
Rods (10^3^ mm/µL)	0.00 ± 0.00	0.00 ± 0.00	0.00 ± 0.00
Segmented (10^3^ mm/µL)	2.52 ± 0.32	2.29 ± 0.18	1.97 ± 0.18
Lymphocyte (10^3^ mm/µL)	2.50 ± 0.31	2.23 ± 0.26	1.96 ± 0.11
Monocyte (10^3^ mm/µL)	0.01 ± 0.01	0.02 ± 0.01	0.01 ± 0.00
Platelets (10^5^ mm/µL)	5.22 ± 0.80	5.30 ± 0.39	5.80 ± 0.25

Values are expressed as mean ± S.E.M., *n* = 6. Data are presented as mean ± S.E.M., *n* = 6. Statistical analysis was performed using one-way ANOVA followed by Tukey’s post hoc test. MCHC: Mean corpuscular hemoglobin concentration; MCH: Mean corpuscular hemoglobin; MCV: Mean corpuscular volume.

## Data Availability

The original contributions presented in this study are included in the article. Further inquiries can be directed to the corresponding author.

## Supplementary Materials

The following supporting information can be downloaded at https://www.mdpi.com/article/10.3390/ph19101552/s1, Supplementary Table S1. Phytochemicals from *Baccharis dracunculifolia* DC. tentatively identified based on mass spectrometry profiles. Rt = relative retention times. Supplementary Table S2. The body weight and relative organ weight of Wistar rats treated with vehicle or *Baccharis dracunculifolia* DC. extract (300 or 2000 mg/kg), orally, in a single dose. Supplementary Figure S1–The structures, mass spectra, and cleavage sites of the main components or main classes, found in the extract of *Bacharris dracunculifolia* DC. Supplementary Figure S2—Identification of apiose in *Baccharis dracunculifolia* DC. (A) Partial GC-MS chromatogram in the region of pentoses. (B) Structures of apiose and derivatives apiitol tetra- and pentaacetate, and (C) mass spectra of peaks 1 and 2 that confirmed the presence of apiose in the extract.

## Abbreviations

The following abbreviations are used in this manuscript:

ALT	Alanine aminotransferase
ANOVA	Analysis of variance
ARRIVE	Animal Research: Reporting of In Vivo Experiments
AST	Aspartate aminotransferase
CE	Crude extract
CONCEA	Brazilian National Council for the Control of Animal Experimentation
DDA	Data-dependent analysis
DPPH	2,2-diphenyl-1-picrylhydrazyl
FRAP	Ferric reducing antioxidant power
GAE	Gallic acid equivalents
GC-MS	Gas chromatography–mass spectrometry
HRBC	Human red blood cell
IC_50_	Half-maximal inhibitory concentration
ISO	International Organization for Standardization
MCH	Mean corpuscular hemoglobin
MCHC	Mean corpuscular hemoglobin concentration
MCV	Mean corpuscular volume
MTT	3-(4,5-dimethylthiazol-2-yl)-2,5-diphenyltetrazolium bromide
NC	Negative control
NL	Neutral losses
OECD	Organisation for Economic Co-operation and Development
PC	Positive control
QE	Quercetin equivalents
SisGen	Brazilian National System for Management of Genetic Heritage and Associated Traditional Knowledge
SQMG	Sulfoquinovosylmonoacylglycerol
TPTZ	2,4,6-tris(2-pyridyl)-s-triazine
UHPLC-QTOF-MS	Ultra-high-performance liquid chromatography coupled with quadrupole time-of-flight mass spectrometry
UNIPAR	Universidade Paranaense
