# Supplementary material for: Phytochemical Characterization and Integrated Biological Evaluation of Baccharis dracunculifolia DC Ethanol Extract: Antioxidant, Anti-Inflammatory and Toxicological Evidence
Source: Pharmaceuticals (Basel). 2026 Sep 30;19(10):1552. doi: 10.3390/ph19101552 (PMC13708211; doi:10.3390/ph19101552)
Supplement: Supplementary file 1 [file pharmaceuticals-19-01552-s001.zip › pharmaceuticals-4547778-supplementary.pdf]

Supplementary Table S1. Phytochemicals from *Baccharis dracunculifolia* DC. tentatively identified based on mass spectrometry profiles. Rt = relative retention times.

Peak	*Rt*	MS[1] (observed m/z)	formula (m/z)	Error (ppm)	MS[2]	Tentative Identification
1	0.84	195.0504	$C_6H_{11}O_7$	-3.4	129.018, 99.008, 87.008, 75.007, 59.013	Gluconic acid
2	0.90	191.0556	$C_7H_{11}O_6$	-2.6	173.045, 127.039, 111.008, 93.033, 85.028	Quinic acid
3	1.09	191.0189	$C_6H_7O_7$	-4	111.008, 87.008, 85.028, 67.018	Isocitric acid
4	1.35	191.0191	$C_6H_7O_7$	-3.1	111.008, 87.008, 85.028, 67.018	Citric acid
5	4.48	353.1448 (399.1499)	$C_{14}H_{25}O_{10}$ (HCO_2)-	-1.5	221.103, 161.045, 113.024, 101.023	Propyl acuminoside/apiinibioside
6	5.51	219.0502	$C_8H_{11}O_7$	-3.9	111.008, 87.008, 67.018	Citric acid dimethyl ester
7	6.50	353.0872	$C_{16}H_{17}O_9$	-1.6	191.055, 179.034, 135.044	Chlorogenic acid (isomer 1)
8	6.77	447.1136	$C_{18}H_{23}O_{13}$	-1.5	315.073, 153.018, 152.011, 109.028, 108.021	Dihydroxybenzoic acid acuminoside/apiinibioside
9	7.09	315.1077	$C_{14}H_{19}O_8$	-2.6	195.065, 151.075, 125.023, 83.013	Hydroxytyrosol-hexoside
10	8.71	417.1031	$C_{17}H_{21}O_{12}$	-1.8	285.062, 241.071, 153.019, 152.011, 109.028, 108.020	Dihydroxybenzoic acid dipentoside
11	9.36	353.0875	$C_{16}H_{17}O_9$	-1	191.055, 179.034, 173.044, 135.044	Chlorogenic acid (isomer 2)
12	10.02	401.1444 (447.1500)	$C_{18}H_{25}O_{10}$	-2.2	293.088, 269.102, 233.066, 161.045, 113.023, 101.023	Benzyl acuminoside/apiinibioside
13	10.20	239.059	$C_8H_{15}O_6S$	-2.4	159.102, 113.096, 96.959, 79.956	Sulfated compound
14	10.63	381.1757 (427.1814)	$C_{16}H_{29}O_{10}$	-2.2	293.089, 249.134, 161.045, 113.024, 101.024	Isopentyl acuminoside/apiinibioside
15	11.57	593.1514	$C_{27}H_{29}O_{15}$	0.3	503.118, 473.108, 413.087, 383.076, 353.066	Vicenin 2
16	11.88	385.1134	$C_{17}H_{21}O_{10}$	-1.6	267.072, 249.061, 207.051, 135.044, 113.023, 91.054	Phenylacetyl glucuronosylglycerol
17	12.35	563.1398	$C_{26}H_{27}O_{14}$	-1.5	503.119, 473.108, 443.096, 383.077, 353.066	Vicenin (isomer 1)
18	12.67	563.1410	$C_{26}H_{27}O_{14}$	0.7	503.119, 473.108, 443.096, 383.077, 353.066	Vicenin (isomer 2)
19	13.05	519.1725	$C_{22}H_{31}O_{14}$	1.1	399.129, 339.108, 309.097, 279.087, 237.076, 209.045, 167.034	Dihydroferulic acid dihexoside
20	13.19	563.1398	$C_{26}H_{27}O_{14}$	-1.5	503.119, 473.108, 443.096, 383.077, 353.066	Vicenin (isomer 3)

21	13.33	563.1398	$C_{26}H_{27}O_{14}$	-1.5	473.108, 443.097, 383.077, 353.066	Vicenin (isomer 4)
22	13.42	479.2489	$C_{22}H_{39}O_{11}$	-1.8	179.055, 161.044, 143.034, 119.034, 113.023, 101.023, 89.023	Dihexosyl-citronellol
23	13.42	741.1871	$C_{32}H_{37}O_{20}$	-1.8	621.183, 577,193, 301.035, 300.027	Quercetin triglycoside
24	13.42	755.1820	$C_{36}H_{35}O_{18}$	-1.2	593.150, 575.140, 473.109, 455.097, 353.064, 179.034, 135.043	Vicenin-2 caffeoate
25	14.40	477.0667	$C_{21}H_{17}O_{13}$	-1.5	301.035, 255.029, 178.998, 151.003	Quercetin glucoronide
26	14.51	463.0874	$C_{21}H_{19}O_{12}$	-1.8	301.035/300.027, 271.024, 255.029, 178.997, 151.003	Quercertin hexoside
27	15.07	515.1198	$C_{25}H_{23}O_{12}$	0.6	353.087, 335.077, 191.055, 179.034, 173.045, 135.044	Dicafeoylquinic acid (isomer 1)
28	15.56	515.1194	$C_{25}H_{23}O_{12}$	-0.2	353.087, 191.055, 179.034, 135.044	Dicafeoylquinic acid (isomer 2)
29	16.40	515.1198	$C_{25}H_{23}O_{12}$	0.6	353.087, 191.055, 179.034, 173.045, 135.044	Dicafeoylquinic acid (isomer 3)
30	17.24	377.1599	$C_{20}H_{25}O_{7}$	-1.7	329.138, 289.180, 259.170, 241.160, 125.024	Clerodane diterpene
31	17.36	379.1756	$C_{20}H_{27}O_{7}$	-1.7	361.165, 335.186, 317.175, 305.176, 287.165, 261.186, 243.175, 241.159, 221.118, 201.127, 177.127,155.086	Clerodane diterpene
32	17.48	379.1755	$C_{20}H_{27}O_{7}$	-1.8	361.165, 335.186, 317.176, 305.174, 287.164, 261.185, 243.175, 241.159, 223.149, 213.164, 155.085,126.031	Clerodane diterpene
33	17.65	379.1756	$C_{20}H_{27}O_{7}$	-1.7	361.165, 331.155, 291.196, 287.165, 261.185, 243,175	Clerodane diterpene
34	18.50	377.1598	$C_{20}H_{25}O_{7}$	-2	329.138, 289.180, 259.170, 241.160, 125.024	Clerodane diterpene
35	19.05	207.0656	$C_{11}H_{11}O_{4}$	-3.5	179.034, 161.023, 135.044, 133.028	Ethyl caffeoate
36	19.67	361.1651	$C_{20}H_{25}O_{6}$	-1.6	317.176, 287.165, 243.175, 155.086	Clerodane diterpene
37	19.95	361.1650	$C_{20}H_{25}O_{6}$	-1.8	317.175, 287.165, 243.175, 126.031	Clerodane diterpene
38	19.97	677.1504	$C_{34}H_{29}O_{15}$	-1.1	515.119, 353.087, 335.076, 191.055, 179.034, 173.045	Tricaffeoyl quinic acid
39	20.16	363.1809	$C_{20}H_{27}O_{6}$	-1	345.171, 319.191, 289.180, 275.201, 245.190, 243.175, 159.117	Clerodane diterpene

40	20.42	363.1809	$C_{20}H_{27}O_6$	-1	345.171, 319.191, 289.180, 275.201, 245.190, 243.175, 159.117	Clerodane diterpene
41	21.42	345.1337	$C_{19}H_{21}O_6$	-1.8	301.144, 257.154, 227.143, 227.143, 185.096, 175.112, 171.081, 157.065	Clerodane diterpene
42	21.58	345.1338	$C_{19}H_{21}O_6$	-1.7	301.144, 257.154, 219.102, 213.127, 187.757, 175.112, 159.081	Clerodane diterpene
43	21.76	299.0559	$C_{16}H_{11}O_6$	-0.6	284.032, 255.029, 227.034, 212.047, 183.044, 136.987	Kaempferide
44	22.99	925.4797	$C_{47}H_{73}O_{18}$	-0.6	775.428, 763.425, 701.427, 613.374, 569.384, 523.379, 455.351	Saponin triglycoside
45	24.20	485.2904	$C_{29}H_{41}O_6$	-1	455.279, 437.269, 317.175, 163.039, 145.029, 119.049, 117.034	Terpene
46	24.51	389.1964	$C_{22}H_{29}O_6$	-1.6	359.185, 315.196, 287.165, 285.149, 269.154, 243.175	Terpene
47	24.70	391.2122	$C_{22}H_{31}O_6$	-1	362.173, 345.170, 332.163, 331.155, 271.170, 243.175, 205.123, 111.008	Terpene
48	25.80	721.3640 $[M+HCO_2]^-$	$C_{33}H_{55}O_{14}$ (HCO_2)	-1.7	675.3596, 415.145, 397.135, 277.216, 235.082, 179.055, 161.046	Digalactosylmonoacylglycerol ($C_{18:3}$)
49	26.20	577.2686	$C_{27}H_{45}O_{11}S$	-0.3	317.053, 299.043, 277.216, 225.007, 206.996, 164.985, 152.985, 94.980	Sulfoquinovosylmonoacylglycerol ($C_{18:3}$)
50	26.94	299.1644	$C_{19}H_{23}O_3$	-2.9	255.175, 244.109, 200.120, 185.096, 145.065	Artepillin C
51	27.06	559.3114 $[M+HCO_2]^-$	$C_{27}H_{45}O_9$ (HCO_2)	-1.8	277.215, 253.092, 235.082, 179.056, 161.045	Monogalactosylmonoacylglycerol ($C_{18:3}$)
52	27.06	699.3797 $[M+HCO_2]^-$	$C_{31}H_{57}O_{14}$	-1.7	653.373, 415.145, 397.135, 305.087, 255.232, 235.082, 179.055	Digalactosylmonoacylglycerol ($C_{16:0}$)
53	27.14	579.2832	$C_{27}H_{47}O_{11}S$	-1.6	299.043, 279.233, 225.007, 206.996, 164.985, 152.985, 94.980	Sulfoquinovosylmonoacylglycerol ($C_{18:2}$)
54	27.72	555.2847	$C_{25}H_{47}O_{11}S$	0.4	317.053, 299.043, 255.232, 225.007, 206.996, 164.985, 152.985, 94.980	Sulfoquinovosylmonoacylglycerol ($C_{16:0}$)

Supplementary Table S2. The body weight and relative organ weight of Wistar rats treated with vehicle or *Baccharis dracunculifolia* DC. extract (300 or 2000 mg/kg), orally, in a single dose.

**Day**	**Basal**	**Extract of *Baccharis dracunculifolia***	
		**300 mg/kg**	**2000 mg/kg**
		Body weight (g)	
0	215.60 ± 1.79	201.90 ± 2.92	193.10 ± 3.38
1	216.20 ± 1.93	202.70 ± 3.53	193.80 ± 3.77
2	216.30 ± 2.04	203.50 ± 3.60	193.70 ± 3.73
3	218.70 ± 1.82	204.00 ± 3.96	196.00 ± 3.56
4	221.50 ± 1.91	206.30 ± 4.12	198.80 ± 3.51
5	223.20 ± 1.96	207.50 ± 3.83	199.30 ± 3.93
6	224.50 ± 2.51	210.50 ± 3.90	202.70 ± 3.62
7	225.50 ± 2.26	212.00 ± 3.16	206.00± 3.83
8	227.20 ± 2.16	214.70 ± 3.49	209.00 ± 4.25
9	229.80 ± 2.16	216.50 ± 3.77	212.20 ± 4.82
10	230.70 ± 1.83	219.00 ± 3.73	214.20 ± 3.90
11	223.00 ± 2.35	222.50 ± 3.04	216.00 ± 3.80
12	232.50 ± 2.34	222.20 ± 3.52	217.00 ± 3.79
13	235.50 ± 1.66	225.20 ± 3.38	219.50 ± 4.28
14	236.30 ± 1.89	228.70 ± 3.77	221.50 ± 4.26
		Relative organ weight (%)	
Liver	3.80 ± 0.069	2.75 ± 0.072	2.69 ± 0.091
Spleen	0.19 ± 0.005	0.18 ± 0.003	0.20 ± 0.006
Heart	0.25 ± 0.004	0.28 ± 0.013	0.27 ± 0.009
Kidneys	0.64 ± 0.009	0.60 ± 0.011	0.61 ± 0.018

Day 0 represents baseline body weight measured before extract administration. Values are expressed as mean ± S.E.M., n = 6. Data are presented as mean ± S.E.M., n = 6. Statistical analysis was performed using one-way ANOVA followed by Tukey's post hoc test.

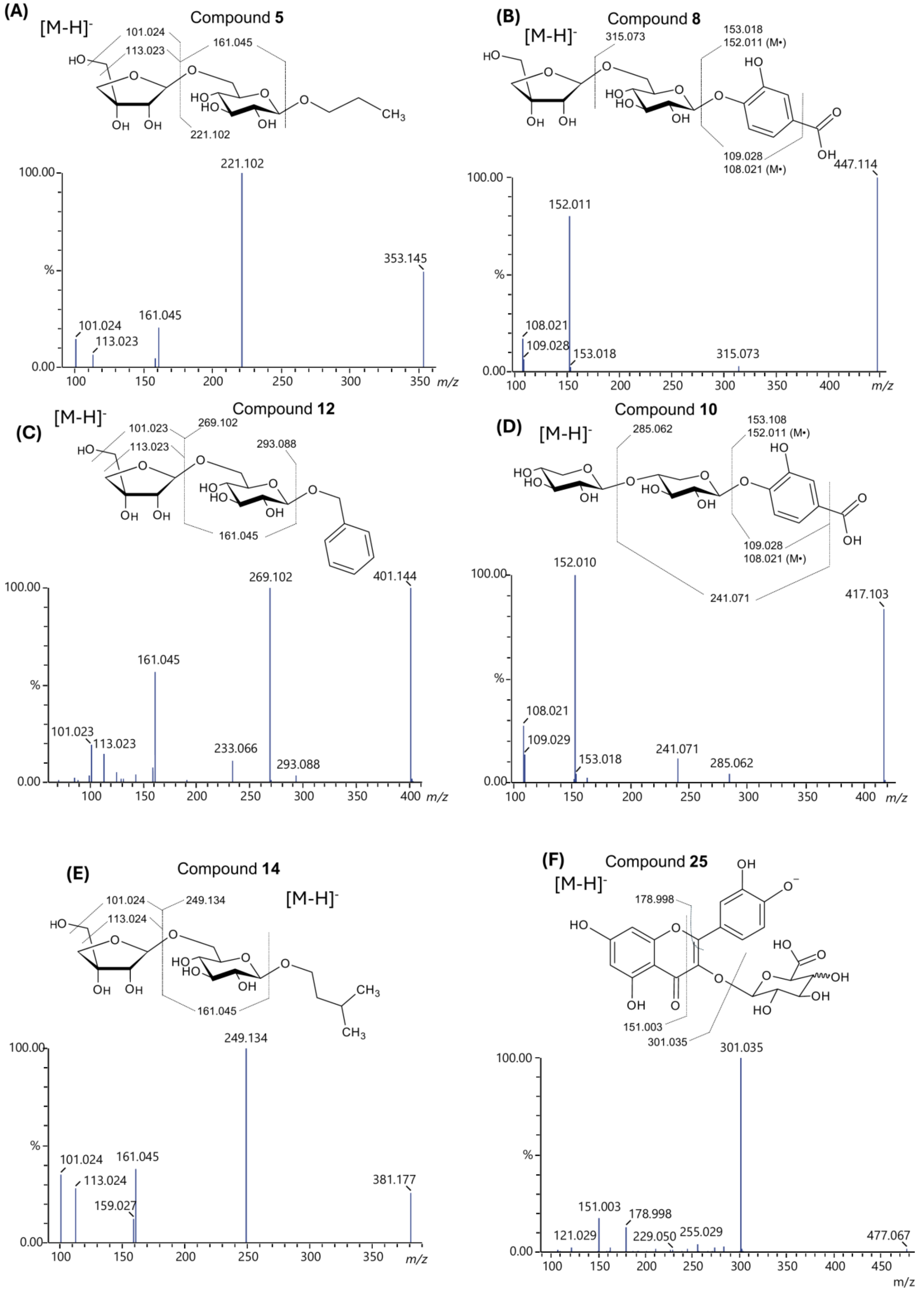
(A)
[M-H]-
Compound 5
101.024
113.023
161.045
221.102
221.102
353.145
161.045
101.024
113.023
(B)
[M-H]-
Compound 8
315.073
153.018
152.011 (M•)
109.028
108.021 (M•)
447.114
152.011
108.021
109.028
153.018
315.073
(C)
[M-H]-
Compound 12
101.023
113.023
269.102
293.088
161.045
269.102
401.144
161.045
101.023
113.023
233.066
293.088
(D)
[M-H]-
Compound 10
285.062
153.108
152.011 (M•)
109.028
108.021 (M•)
241.071
152.010
417.103
108.021
109.029
153.018
241.071
285.062
(E)
Compound 14
[M-H]-
101.024
113.024
249.134
161.045
249.134
101.024
161.045
113.024
159.027
381.177
(F)
Compound 25
[M-H]-
178.998
151.003
301.035
301.035
151.003
178.998
121.029
229.050
255.029
477.067
100.00
%
0.00
m/z

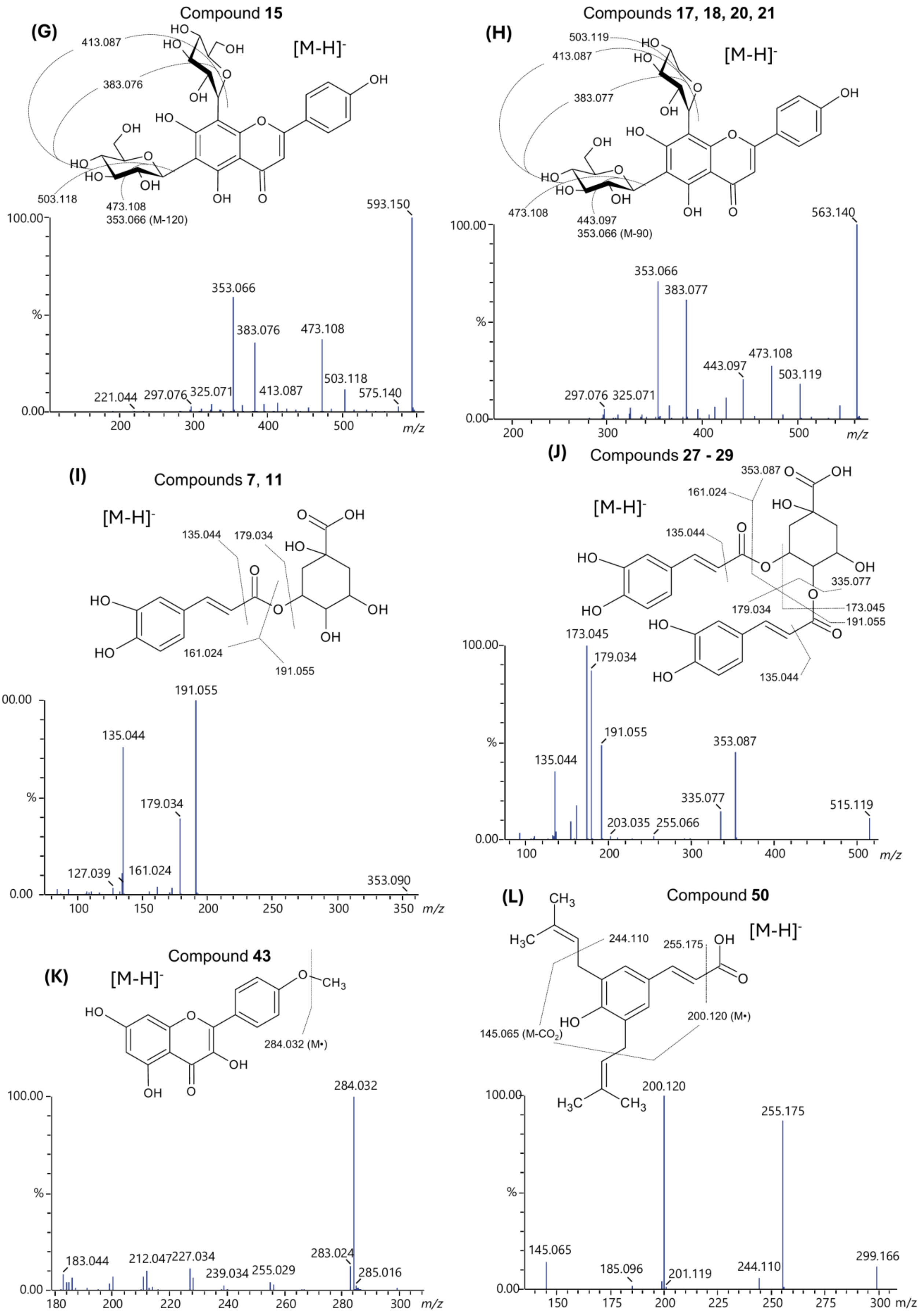
(G)
Compound 15
[M-H]-
413.087
383.076
503.118
473.108
353.066 (M-120)
593.150
353.066
383.076
473.108
413.087
503.118
575.140
221.044
297.076
325.071
100.00
%
0.00
200
300
400
500
m/z
(H)
Compounds 17, 18, 20, 21
[M-H]-
503.119
413.087
383.077
473.108
443.097
353.066 (M-90)
563.140
353.066
383.077
473.108
443.097
503.119
297.076
325.071
100.00
%
0.00
200
300
400
500
m/z
(I)
Compounds 7, 11
[M-H]-
135.044
179.034
161.024
191.055
191.055
135.044
179.034
127.039
161.024
353.090
100.00
%
0.00
100
150
200
250
300
350
m/z
(J)
Compounds 27 - 29
[M-H]-
353.087
161.024
135.044
335.077
179.034
173.045
191.055
135.044
173.045
179.034
191.055
135.044
353.087
335.077
515.119
203.035
255.066
100.00
%
0.00
100
200
300
400
500
m/z
(K)
Compound 43
[M-H]-
284.032 (M•)
284.032
183.044
212.047
227.034
239.034
255.029
283.024
285.016
100.00
%
0.00
180
200
220
240
260
280
300
m/z
(L)
Compound 50
[M-H]-
244.110
255.175
145.065 (M-CO2)
200.120 (M•)
200.120
255.175
145.065
185.096
201.119
244.110
299.166
100.00
%
0.00
150
175
200
225
250
275
300
m/z

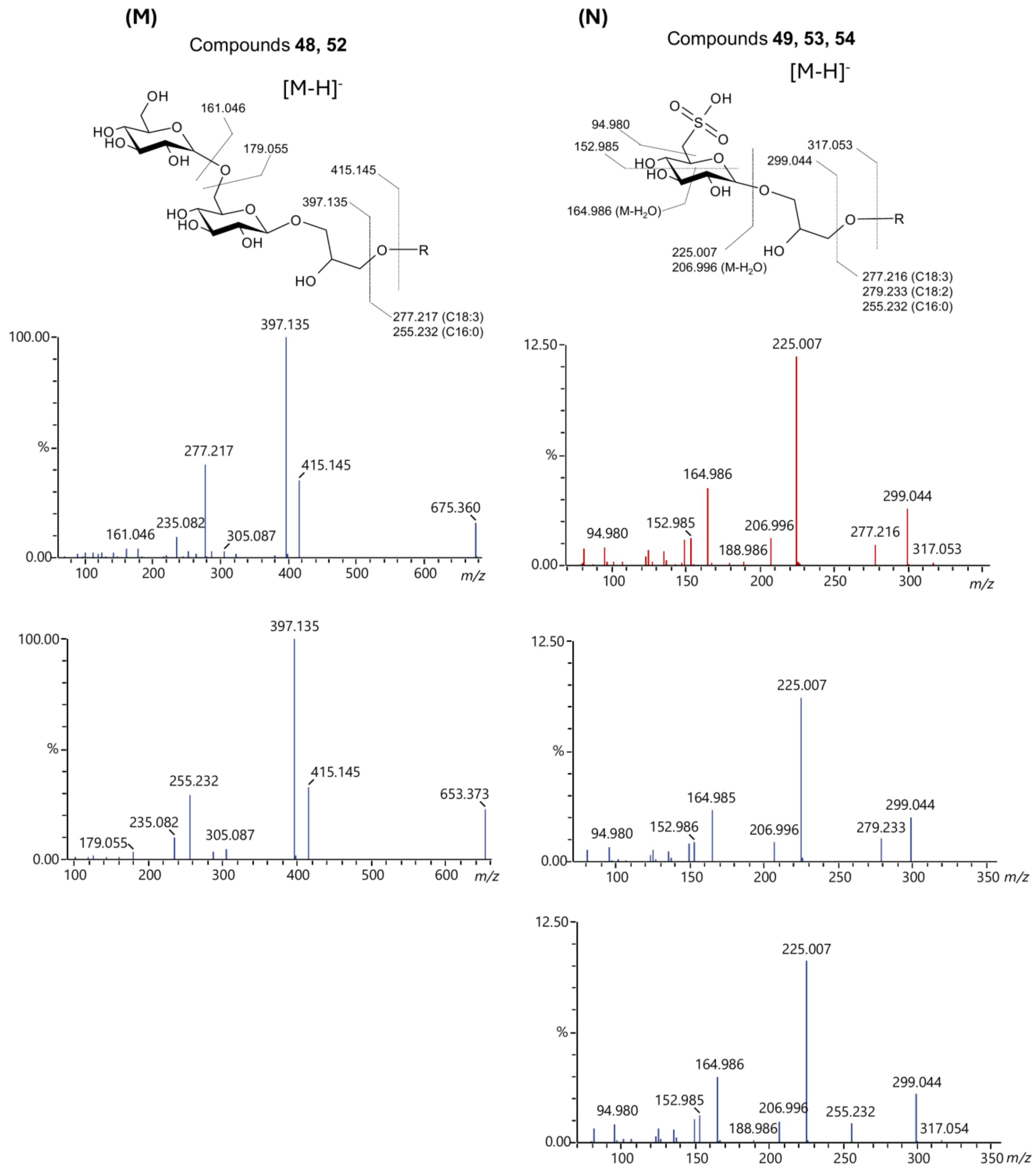

Supplementary Figure S1– The structures, mass spectra, and cleavage sites of the main components or main classes, found in the extract of *Bacharris dracunculifolia* DC.

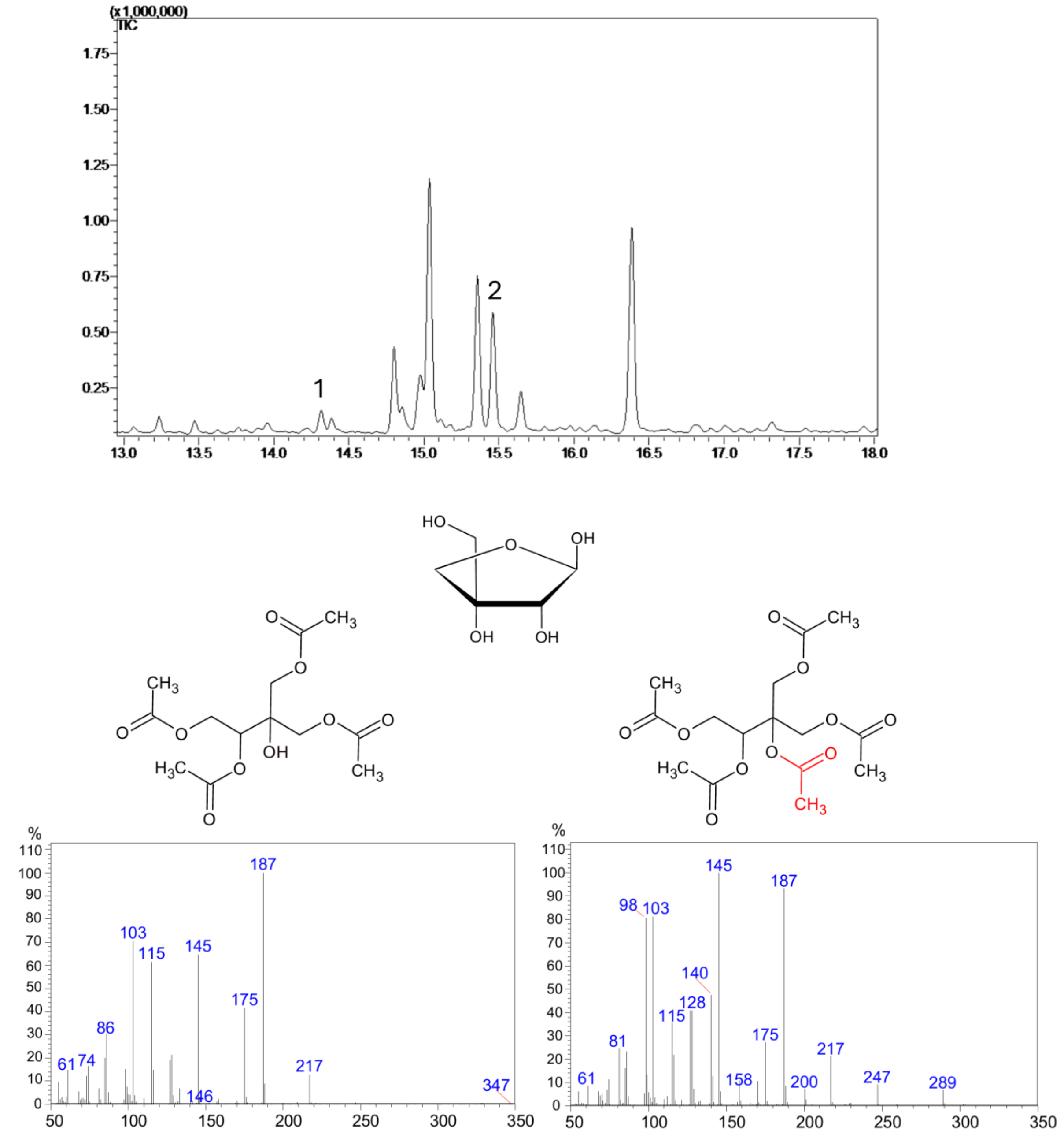

Supplementary Figure S2 – Identification of apiose in *Baccharis dracunculifolia* DC. (A) Partial GC-MS chromatogram in the region of pentoses. (B) Structures of apiose and derivatives apiitol tetra- and pentaacetate, and (C) mass spectra of peaks 1 and 2 that confirmed the presence of apiose in the extract.
